# Physiological and Biochemical Traits of Two Major *Arabidopsis* Accessions, Col-0 and Ws, Under Salinity

**DOI:** 10.3389/fpls.2021.639154

**Published:** 2021-06-21

**Authors:** Maïté Leschevin, Marwa Ismael, Anthony Quero, Hélène San Clemente, Romain Roulard, Solène Bassard, Paulo Marcelo, Karine Pageau, Elisabeth Jamet, Catherine Rayon

**Affiliations:** ^1^UMR INRAE 1158 BioEcoAgro, BIOlogie des Plantes et Innovation, Université de Picardie Jules Verne, Amiens, France; ^2^LRSV, Université de Toulouse, CNRS, UPS, Auzeville-Tolosane, France; ^3^Plateforme d’Ingénierie Cellulaire & Analyses des Protéines ICAP Université de Picardie Jules Verne, Amiens, France

**Keywords:** antioxidant enzymes, *Arabidopsis*, cell wall, metabolites, pigments, salt stress, TMTs quantitative proteomics

## Abstract

Salinity affects plant growth and development as shown with the glycophyte model plant, *Arabidopsis thaliana (Arabidopsis)*. Two *Arabidopsis* accessions, Wassilewskija (Ws) and Columbia (Col-0), are widely used to generate mutants available from various *Arabidopsis* seed resources. However, these two ecotypes are known to be salt-sensitive with different degrees of tolerance. In our study, 3-week-old Col-0 and Ws plants were treated with and without 150 mM NaCl for 48, 72, or 96 h, and several physiological and biochemical traits were characterized on shoots to identify any specific traits in their tolerance to salinity. Before salt treatment was carried out, a different phenotype was observed between Col-0 and Ws, whose main inflorescence stem became elongated in contrast to Col-0, which only displayed rosette leaves. Our results showed that Col-0 and Ws were both affected by salt stress with limited growth associated with a reduction in nutrient uptake, a degradation of photosynthetic pigments, an increase in protein degradation, as well as showing changes in carbohydrate metabolism and cell wall composition. These traits were often more pronounced in Col-0 and occurred usually earlier than in Ws. Tandem Mass Tags quantitative proteomics data correlated well with the physiological and biochemical results. The Col-0 response to salt stress was specifically characterized by a greater accumulation of osmoprotectants such as anthocyanin, galactinol, and raffinose; a lower reactive oxygen detoxification capacity; and a transient reduction in galacturonic acid content. Pectin degradation was associated with an overaccumulation of the wall-associated kinase 1, WAK1, which plays a role in cell wall integrity (CWI) upon salt stress exposure. Under control conditions, Ws produced more antioxidant enzymes than Col-0. Fewer specific changes occurred in Ws in response to salt stress apart from a higher number of different fascilin-like arabinogalactan proteins and a greater abundance of expansin-like proteins, which could participate in CWI. Altogether, these data indicate that Col-0 and Ws trigger similar mechanisms to cope with salt stress, and specific changes are more likely related to the developmental stage than to their respective genetic background.

## Introduction

Salt stress, which results from natural salt accumulation and inappropriate agricultural practices, is a worldwide environmental issue that affects plant growth and development (Munns and Tester, 2008; FAO, 2018). It impairs plant growth by first inducing osmotic stress, due to the excess of salt in the soil resulting in an increased soil osmotic potential, thus disrupting water uptake by roots (Roy et al., 2014). The second phase is ion imbalance, caused by the accumulation of toxic ions such as sodium (Na^+^) and chloride (Cl^–^), leading to calcium (Ca^2+^) and potassium (K^+^) uptake deficiency within the plant (Munns and Tester, 2008; Sun et al., 2009; Almeida et al., 2017). Furthermore, salt stress is accompanied by an oxidative stress characterized by an accumulation of reactive oxygen species (ROS) (Miller et al., 2010). ROS accumulation affects the apoplast, membrane integrity by lipid peroxidation, and the redox regulation of proteins and may even damage DNA (Apel and Hirt, 2004; Møller et al., 2007; Triantaphylidès et al., 2008). All the major plant physiological and biochemical processes are impaired, including photosynthesis mainly characterized by an alteration of chloroplast structure, loss of chlorophyll, and reduction of CO_2_ uptake due to stomatal closing (Chaves et al., 2009; Stepien and Johnson, 2009; Gao et al., 2019).

The changes induced by salt have consequences on carbon partitioning and allocation (Wang et al., 2013; Dong et al., 2018). For instance, in *Arabidopsis thaliana (Arabidopsis)*, the overall allocation is inhibited, and carbon partitioning is reduced into starch and increased into sugars (Kempa et al., 2008; Dong et al., 2018). Soluble sugars can act as osmoprotectant metabolites, but also as signaling molecules that modulate the expression of genes important for salt tolerance (Seifert, 2004; Gong et al., 2005; Kempa et al., 2008; Sun et al., 2013). Most of the carbon fixed by plants during photosynthesis is incorporated into cell wall polysaccharides (Seifert, 2004). The cell wall that is mostly composed of cellulose, hemicelluloses, and pectin (Carpita and Gibeaut, 1993) is also altered during exposure to salt stress as it accumulates salt when vacuoles cannot store it anymore, thereby inhibiting growth (Le Gall et al., 2015). The loss of function of several cell wall–related genes [*UDP-arabinose 4-epimerase 1* (*AtMUR4), pectin methylesterase 31* (*AtPME31*)] or the overexpression of an expansin (*AtEXP3*) or a xyloglucan endotransglucosylase-hydrolase (*AtXTH30*) gene increases salt sensitivity (Kwon et al., 2008; Yan et al., 2018, 2019; Zhao et al., 2019). Meanwhile, the loss of function of *AtXTH30* leads to salt tolerance. The maintenance of cell wall integrity (CWI) during salt stress is important in plant salt tolerance. The module composed of leucine-rich repeat extensins (LRX3/4/5), rapid alkalinization factors (RALF22/23), and the Feronia plasma membrane receptor kinase (FER) participate in CWI under salt stress (Feng et al., 2018; Zhao et al., 2018).

As an adaptive response to salt stress, plants have developed several defense mechanisms (Acosta-Motos et al., 2017). One of them is ion homeostasis to reduce shoot Na^+^ accumulation, by retrieval of Na^+^ from the xylem stream to parenchyma cells through the plasma membrane protein high-affinity potassium transporter 1 (HKT1) (Mäser et al., 2002; Berthomieu et al., 2003; Davenport et al., 2007; Horie et al., 2009). Sodium exclusion is also conducted by the salt overly sensitive signaling pathway (SOS), which increases the Na^+^ efflux from cytosol to apoplast. Incoming Na^+^, in roots and shoots, is stored in the vacuole or in vesicles via Na^+^/H^+^ exchanger proteins (NHX) (Bassil et al., 2011; Maathuis et al., 2014; for a review, see Almeida et al., 2017). Other protective mechanisms including accumulation of osmolytes [glycine betaine and proline (Pro)], polyols (mannitol, sorbitol, and galactinol), and sugars (sucrose, glucose, raffinose, trehalose, etc.) maintain ion homeostasis and stabilize cellular and protein structures (Liu and Zhu, 1997; Nishizawa et al., 2008; Sun et al., 2013; Gupta and Huang, 2014; Gao et al., 2019; Sharma et al., 2019). ROS scavenging also plays a crucial role in plant salt stress tolerance (Slama et al., 2015). Plants possess a very efficient enzymatic system that produces H_2_O_2_ by superoxide dismutase (SOD; EC 1.15.1.1), which is then detoxified by catalase (CAT; EC 1.11.1.6) or the ascorbate–glutathione cycle. This cycle consists of ascorbate peroxidase (APX; EC 1.11.1.11), monodehydroascorbate reductase (MDHAR; EC 1.6.5.4), dehydroascorbate reductase (DHAR; EC:1.8.5.1), and glutathione reductase (GR; EC 1.6.4.2), which reduce non-enzymatic antioxidants (ascorbic acid, and glutathione) (Ahanger et al., 2017; Shafi et al., 2019). Polyamines (spermine, spermidine, and putrescine) also contribute to ROS homeostasis during salt stress by enhancing plasma membrane stability; promoting CAT, SOD, and peroxidase activities; and modulating H^+^/ATPase and Ca^2+^/ATPase transporters (Shabala et al., 2006; Kusano et al., 2008; Roychoudhury et al., 2011; Bose et al., 2014; Saha et al., 2015; Zapata et al., 2017). Secondary metabolites including pigments, such as carotenoids (Krinsky, 1979; Ruiz-Sola et al., 2014; Brunetti et al., 2015) or anthocyanin (Eryılmaz, 2006; van Oosten et al., 2013; Kovinich et al., 2014), are also involved in ROS detoxification.

Environmental pressures lead plants to local adaptation and to generate natural phenotypic and genetic variations. Because of its widespread geographic distribution, *Arabidopsis* experiences a broad range of climatic conditions (Mitchell-Olds and Schmitt, 2006). Therefore, many *Arabidopsis* accessions are available, and some of them are salt-tolerant (Bu-5, Bur-0, Ll-1, Wl-0, Ts-1, and Tsu-1) when compared to Col-0 (Rus et al., 2006; Baxter et al., 2010; Katori et al., 2010; Julkowska et al., 2017). However, *Arabidopsis* is a glycophyte (Kosová et al., 2013) and, surprisingly, is widely explored as a model plant to investigate the molecular mechanism of salt stress tolerance. Indeed, little is known about salinity tolerance of the main reference accessions Wassilewskija (Ws) and Columbia (Col-0), albeit a study reporting that Ws was more salt-tolerant than Col-0, and this was associated with an increase of the expression level of *AtAVP1* encoding a vacuolar H^+^-translocating pyrophosphatase involved in regulating the movement of Na^+^ (Jha et al., 2010). That is surprising as most of the *Arabidopsis* studied mutants are in these well-known genetic backgrounds and available from different seed stock centers.

The aim of this study was to unravel the main differences existing between Col-0 and Ws in their strategy to cope with salt stress. Although root is important to reduce sodium transport into the shoot tissue (Duan et al., 2013; Geng et al., 2013; Julkowska et al., 2014), we have focused our study on shoots because the regulation of salt accumulation in aboveground organs is essential for plant survival.

## Materials and Methods

### Plants Material and Growth Conditions

Two *Arabidopsis* accessions Ws and Col-0 were used. The experiments were performed in three biological replicates with randomized block design. The seeds were surface sterilized by agitation with ethanol 70% containing 0.01% Triton X-100 for 20 min. The seeds were then rinsed with 100% ethanol for 15 min and dried overnight before being resuspended in 500 μL of sterilized water. The seeds were sown on a seed holder containing 0.65% agar. The plants were grown hydroponically in Araponics system (Araponics, Liège, Belgium) using the following Tripack Floraseries media (General Hydroponics Europe, Fleurance, France) adapted from Ward et al. (2011): FloraGrow 0.5 mL ⋅ L^–1^ {3/1/6% N/P/K [1% ammoniacal nitrogen, 2% nitrate nitrogen; 1% available phosphate (P_2_O_5_), 6% soluble potassium (K_2_O), and 0.8% soluble magnesium (MgO)]}; FloraBloom 0.5 mL ⋅ L^–1^ (0/5/4 N/P/K [5% P_2_O_5_, 4% K_2_O, 3% MgO, and 5% soluble sulfur (SO_4_^2–^)]; and FloraMicro 0.5 mL ⋅ L^–1^ {5/0/1% N/P/K [1% ammoniacal nitrogen, 4% nitrate nitrogen, 1.3% K_2_O, 0.01% boron, 7% calcium (CaO), 0.01% copper-chelated EDTA, 0.12% iron-chelated 6% EDDHA-11% DPTA, 0.05% manganese chelated EDTA, 0.002% molybdenum, and 0.015% zinc-chelated EDTA]}, pH 5.5. The seeds were stratified for 2 days at 4°C and grown under controlled conditions (16/8-h light–dark cycle, 120 μmol photons ⋅ m^–2^ ⋅ s^–1^ at 23/19°C). The hydroponic solution was oxygenated by air bubbling and changed once a week. Three-week-old plants were further treated with 0 (control) and 150 mM NaCl for different durations. The shoots were harvested at 48 (T48), 72 (T72), or 96 (T96) h after the beginning of treatment. A total of 50 homogenous plants were harvested per ecotype, time point, biological replicate (*n* = 3), and growing condition (control vs. salt stress). All samples were immediately placed in liquid nitrogen, ground to a fine powder in a ball mill, and stored at −80°C until use.

### Measurement of Fresh and Dry Mass and Shoot Water Content

During the time course of the experiments, shoots were harvested from five plants for each genotype. The fresh mass (FM) was immediately determined. The samples were dried in an oven at 65°C for 3 days to measure the dry masses (DMs). The shoot water content (SWC) and the tolerance index in the shoots (TI) were calculated using the following formula (Jha et al., 2010; Julkowska et al., 2016): SWC (g water/g DM) = (shoot FM − shoot DM)/shoot DM; TI = shoot DM salt/shoot DM control.

### Determination of Ion Content

Ion content in shoots was measured by HPAEC–pulsed amperometry detection (PAD) analysis according to Quéro et al. (2013) using a Dionex ICS-900 Ion Chromatography System (Dionex Corp., Sunnyvale, CA, United States). This device was equipped with an SCS1 (4 × 250 mm) column for cationic solutes or an IonPac AG22 (4 × 50 mm) precolumn and an AS22 (4 × 250 mm) column for anionic solutes and a conductometer DS5. The crude extract (50 μL; §2.6.) was resuspended in 1 mL of ultra-pure water and 10 μL were injected into the column. Cations were separated on the SCS1 (4 × 250 mm) column by isocratic flow at 1.2 mL ⋅ min^–1^ with H_2_SO_4_ (2 mM) as eluent. Anions were separated on the IonPac AG22 precolumn (4 × 50 mm) and the AS22 column (4 × 250 mm) by isocratic flow at 1.2 mL ⋅ min^–1^ with a Na_2_CO_3_ (4.5 mM) and NaHCO_3_ (1.4 mM) buffer as eluent. Data were collected and processed using the Chromeleon 6.50 software (Dionex Corp.). For quantification, calibration ranges were carried out using NaCl, KCl, NaNO_3_, NaH_2_PO_4_, and (NH_4_)_2_SO_4_. The results were expressed in μmol ⋅ g^–1^ DM.

### Pigment Content

Photosynthetic pigments were extracted from 10 mg of freeze-dried shoot powder and quantified according to Duran Garzon et al. (2020). The pigments were separated by reverse-phase high-performance liquid chromatography (Prominence, Shimadzu Co., Kyoto, Japan) using a Zorbax Eclipse PAH column (4.6 × 150 mm, 3.5-μm pore size; Agilent Technologies, Santa Clara, CA, United States) and a UV diode array detector (SPD-M20A; Prominence, Shimadzu Co.) as described by Edelenbos et al. (2001). Commercial pigment standards (neoxanthin, violaxanthin, antheraxanthin, lutein, zeaxanthin, chlorophyll *a* and *b*, and β-carotene) from DHI-Water and Environment (Hørsholm, Denmark) were used for calibration. Canthaxanthin (Sigma-Aldrich, St. Louis, MO, United States) was used as an internal standard for quantification of each pigment, except β-carotene, where a standard curve was performed. Total anthocyanin content was assessed according to Kim et al. (2017). Briefly, 20 mg of frozen leaves was homogenized in 600 μL of an acidified methanol buffer (methanol/HCl 99/1 vol/vol) and extracted under gentle agitation overnight at 4°C in the dark. The anthocyanin extract was purified using 800 μL of water/chloroform (50/50 vol/vol) and centrifuged at 10,000*g* for 5 min. The absorbance of the upper phase was first read at 530 nm and further at 657 nm using a microplate reader (Powerwave; Biotek, Colmar, France). The total anthocyanin content was calculated using the formula A_530_ − 0.25 A_657_, and the result was expressed as corrected A_530_ ⋅ g^–1^ FM (Rabino and Mancinelli, 1986).

### Antioxidant Enzyme Activity Assays

Antioxidant enzyme activity assays are described in Supplementary Material.

### Organic Polar Primary Metabolites Analysis

Primary polar metabolites were extracted from 10 mg of freeze-dried shoot powder as described by Pontarin et al. (2020). Briefly, the sample was homogenized in 400 μL of prechilled methanol supplemented with 200 nmol of ribitol as internal standard and mixed for 10 min at 70°C. Chloroform (200 μL) was added to the reaction and mixed again at 70°C for 5 min. Distilled water (400 μL) was added to the mixture (vortexed for 20 s) before centrifugation (10 min, 10,000*g*). An aliquot of the methanol/water supernatant (50 μL) that constitutes the crude extract was speed-vacuum dried and resuspended in 40 μL of 20 mg ⋅ mL^–1^ methoxyamine hydrochloride in pyridine for 2 h at 37°C. *N*-trimethylsilyl-*N*-methyl trifluoroacetamide (70 μL) was added to the sample and heated at 37°C for 30 min. Gas chromatography–mass spectrometry (GC-MS) analysis was performed on a system composed of a TRACE GC ULTRA gas chromatograph and a DSQII quadrupole mass spectrometer (Thermo Fisher Scientific, Waltham, MA, United States).

### Cell Wall Sugar Composition

Plant cell wall material was prepared from 150 mg of frozen shoot powder as described by Duran Garzon et al. (2020). Dry cell wall material was digested with amylase according to Fleischer et al. (1999). After digestion, the dry cell wall (2 mg) was hydrolyzed, and the samples were injected onto a CarboPac-1 column (Dionex) HPAEC, separated, and detected by PAD. Data were collected and processed using the Chromeleon 6.50 software (Dionex Corp.) (Duran Garzon et al., 2020).

### Proteomics Analysis

Proteins were extracted from the shoots using a global protein extraction method adapted from Ma et al. (2016) and described by Leschevin et al. (2021). Proteins were identified and quantified using a Tandem Mass Tags (TMTs)–based comparative proteomics analysis method (Leschevin et al., 2021). These experiments were performed on three biological replicates. Data were processed using Proteome Discoverer 2.4 (Thermo Fisher Scientific) before being run against The *Arabidopsis* information Resource (TAIR10_pep_20101214), as well as against Swissprot^1^. Proteins were annotated using the *ProtAnnDB* (San Clemente et al., 2009) and Uniprot^2^ databases. The MS/MS data (raw data, identification, and quantification results) are available at ProteomeXchange with identifier PXD022441^3^. To identify differentially accumulated proteins (DAPs), the results were classified according to the ratio of amount of proteins in salt-stressed shoots vs. control shoots for a given ecotype at T48, T72, and T96. We have defined the DAPs as those with a fold change >1.7 or <0.6 at a *p* < 0.05 between two comparison groups (T48, T72, and T96).

### Statistical Analyses

Statistical analyses were performed using Kruskal–Wallis test (Kruskal and Wallis, 1952). Multiple comparisons after the Kruskal–Wallis test were performed using *post hoc* Dunn test (Dunn, 1964). A significance threshold of 0.05 was applied in all tests. R version 4.0.2 was used for all analyses.

## Results

### Physiological Assessment of Ws and Col-0 Salt-Treated Plants

**FIGURE 1:**
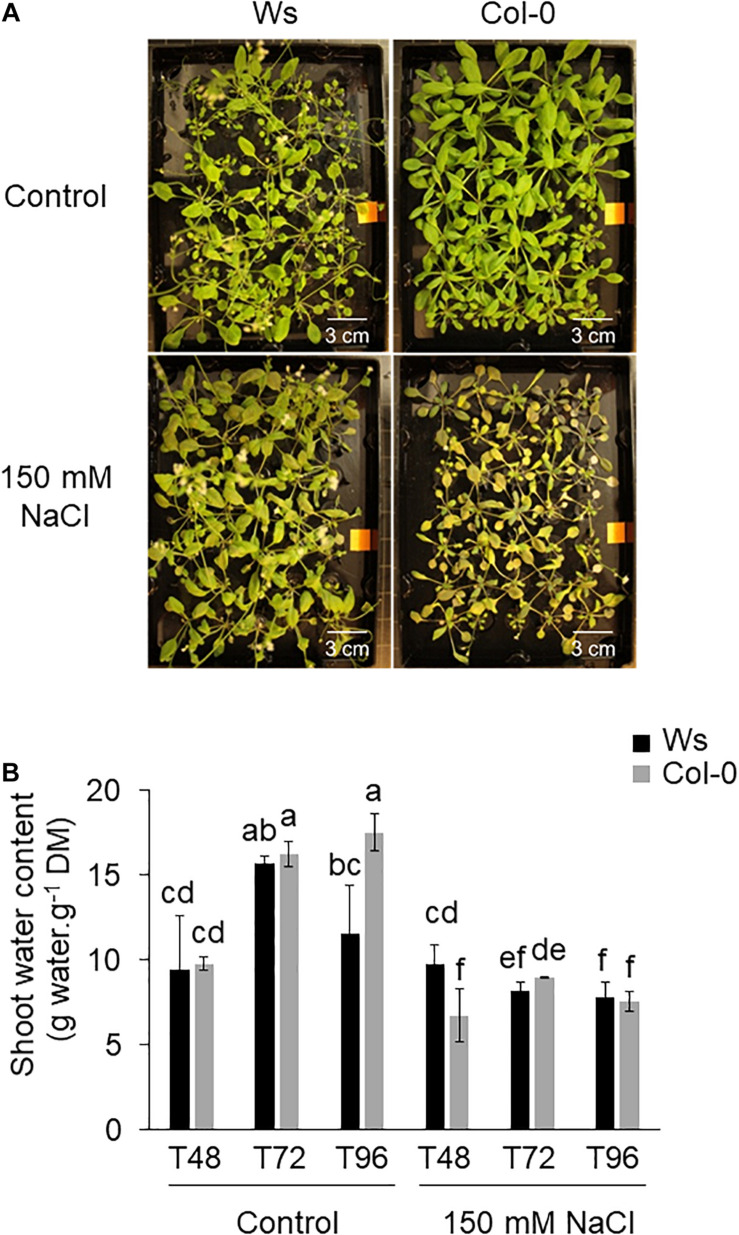
**(A)** Shoot phenotype of *Arabidopsis* ecotypes Wassilewskija (Ws) and Columbia (Col-0) in response to salt stress. The 3-week-old plants grown hydroponically without (control) or in the presence of 150 mM of NaCl are shown at T96. **(B)** Effect of salt stress on the shoot tolerance index in Ws and Col-0 salt-treated plants. Data are means ± standard deviation of three biological replicates. Means followed by the same letter in the same graph are not significantly different according to Dunn test.

To determine the natural variation between Col-0 and Ws, control and salt-treated plants were phenotyped by measuring different parameters including the FM of the shoots, the SWC, and the tolerant index of the shoots (TI). Under control conditions, Col-0 only showed rosette leaves, whereas Ws already produced the main inflorescence stem (Figure 1A). A decrease in FM was observed in both ecotypes after a prolonged salt stress exposure (T72 and T96) (Supplementary Figure 1A). This reduction in shoot biomass was associated with a significantly lower SWC and TI, which decreased similarly in Ws and Col-0 for each time point (Figure 1B and Supplementary Figure 1B).

### The Mineral Status in Col-0 and Ws Shoots Under Salt Stress

The macronutrients content including Na^+^, K^+^, Cl^–^, NO_3_^–^, and SO_4_^2–^ was determined in the shoots of Ws and Col-0. Na^+^ and Cl^–^ concentrations quickly reached toxic levels in both ecotypes under salt treatment with a Na^+^ increased level by nearly 33-fold (Ws) and 36-fold (Col-0) at T48, 62-fold (Ws), and 66-fold (Col-0) at T72, and 48-fold (Ws) and 45-fold (Col-0) at T96 (Table 1), indicating that the capacity to accumulate Na^+^ into their shoots was similar in both ecotypes. In a similar manner, the content of Cl^–^ accumulated in both ecotypes in response to salt stress. However, this was in a range of 1.6-fold greater in Ws at T48 (95-fold) and T72 (136-fold) as compared to Col-0 (59-fold at T48 and 82-fold at T72), before reaching a similar trend at T96 (67-fold in Col-0 vs. 74-fold in Ws). This suggests that the Cl^–^ uptake and its translocation to shoots are higher in Ws than in Col-0 under high salinity.

**TABLE 1:** Elemental composition (Na^+^, K^+^, Cl^–^, NO_3_^–^, and SO_4_^2–^) (μmol ⋅ g^–1^ DM) of the shoots of Ws and Col-0 plants grown under control conditions or 150 mM NaCl during T48, T72, and T96.

**μmol ⋅ g^–1^ DM**		**Na^+^**	**K^+^**	**Cl^–^**	**NO_3_^–^**	**SO_4_^2–^**
Ws control	T48	80.1 ± 11.1^de^	1,115.9 ± 33.7^a^	24.9 ± 1.2^e^	1,443.6 ± 445.8^ab^	56.3 ± 11.0^a^
	T72	54.5 ± 39.1^e^	1,022.7 ± 4.9^ab^	21.3 ± 8.1^e^	1,356.8 ± 68.4^ab^	49.7 ± 3.6^ab^
	T96	49.0 ± 27.5^e^	1,019.0 ± 5.9^ab^	32.0 ± 10.6^e^	1,025.1 ± 195.4^bcd^	41.7 ± 9.3^abc^
Ws 150 mM NaCl	T48	2,676.6 ± 348.8^ab^	762.5 ± 117.1^cde^	2,367.0 ± 90.5^ab^	660.5 ± 80.5^cde^	28.1 ± 9.4^def^
	T72	3,375.6 ± 572.1^a^	694.3 ± 12.1^cde^	2,930.5 ± 443.0^a^	521.0 ± 106.1^def^	32.6 ± 3.3^cde^
	T96	2,346.3 ± 414.7^*bc*^	475.4 ± 8.1^fg^	2,352.1 ± 489.0^ab^	214.1 ± 59.3^*f*^	19.9 ± 3.0^ef^
Col-0 control	T48	93.1 ± 1.1^d^	886.6 ± 73.8^bc^	25.6 ± 0.1^e^	1,134.1 ± 48.2^*bc*^	38.6 ± 8.4^abcd^
	T72	34.7 ± 5.2^e^	973.0 ± 214.4^ab^	25.5 ± 4.6^e^	1,597.7 ± 184.0^a^	68.4 ± 38.2^ab^
	T96	54.81 ± 34.0^e^	855.07 ± 3.3^bc^	30.08 ± 6.0^e^	1,313.8 ± 35.1^ab^	36.0 ± 1.8^bcd^
Col-0 150 mM NaCl	T48	2,544.8 ± 872.2^a^	647.9 ± 30.9^def^	1,509.5 ± 529.2^bcd^	358.1 ± 326.8^ef^	18.0 ± 7.4^ef^
	T72	2,288.3 ± 72.9^*bc*^	612.6 ± 7.2^efg^	2,084.2 ± 76.4^ab^	344.4 ± 213.4^ef^	20.8 ± 8.4^ef^
	T96	2,457.3 ± 272.0^abc^	451.5 ± 21.8^g^	2,018.7 ± 224.4^abc^	178.3 ± 24.5^*f*^	15.0 ± 2.8^f^

*Data are means ± standard deviation of three biological replicates. Means followed by the same letter in the same column are not significantly different according to Dunn test.*

As salinity can affect nutrient uptake, we then measured the amounts of K^+^, NO_3_^–^, and SO_4_^2–^. Under control conditions, the other ions (K^+^, NO_3_^–^, and SO_4_^2–^) showed a similar content in both ecotypes. When the plants were exposed to salt stress, a decreased level of K^+^ by nearly twofold was observed at T96 in both ecotypes. The K^+^/Na^+^ ratio greatly dropped under salt stress by 50-fold at T48 and nearly by 100-fold at T72 and T96 in both accessions, confirming that Na^+^ reduces K^+^ uptake in both ecotypes. The NO_3_^–^ content was reduced by two- and threefold at T48 upon salt stress in Ws and Col-0, respectively, and this decrease reached 5.0- and 7.4-fold at T96 in Ws and Col-0, respectively. As a result, the NO_3_^–^/Cl^–^ ratio dramatically dropped by 350- and 500-fold at T96 in Ws and Col-0, respectively. Furthermore, SO_4_^2–^ uptake was reduced by 154- and 161-fold at T96 as shown by the SO_4_^2–^/Cl^–^ ratio in Ws and Col-0, respectively. All these results showed that high levels of Na^+^ and/or Cl^–^ uptake in plants reduce NO_3_^–^ and SO_4_^2–^ translocation to shoots and may have a negative effect on sulfur and nitrogen metabolism in both ecotypes.

### Effect of Salt Stress on Photosynthetic Pigment and Anthocyanin Contents in the Shoots of Ws and Col-0

Salt stress showed a visible leaf pigment alteration phenotype in both ecotypes, albeit stronger in Col-0 (Figure 1A). That was correlated with a reduction in chlorophyll (chl) *a* by 1.5-fold in both ecotypes and in chl *b* more pronounced in Col-0 than in Ws (1.6-fold in Col-0 vs. 1.3-fold in Ws) (Supplementary Figure 2A). The carotenoids content decreased similarly in both Col-0 (1.5-fold) and Ws (1.4-fold) compared with control plants (Supplementary Figure 2B).

**FIGURE 2:**
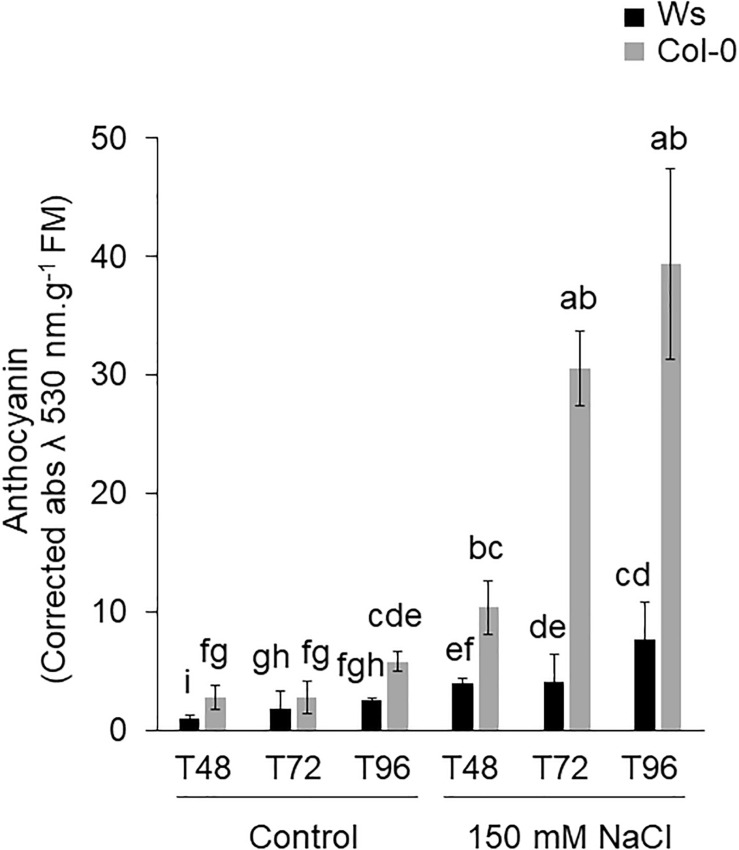
Shoot anthocyanin content of Ws and Col-0 at T48, T72, and T96 under control and 150 mM NaCl conditions. Data are means ± standard deviation of three biological replicates. Means followed by the same letter are not significantly different according to Dunn test.

The anthocyanin content was significantly higher in Col-0 (1.5- to 2.8-fold) than in Ws, under control conditions (Figure 2). Under salt exposure, an increase in anthocyanin was observed in both ecotypes. However, Col-0 produced more anthocyanin than Ws under salinity (Figure 2). Unlike at T48, where a similar increase by approximately fourfold was observed in both ecotypes, Col-0 accumulated more anthocyanin at T72 (sevenfold) and at T96 (fivefold) than Ws, where a two- and threefold increased level was observed at T72 and T96, respectively.

### Antioxidant Enzyme Activities in Ws and Col-0 Shoots After Exposure to Salt Stress

**FIGURE 3:**
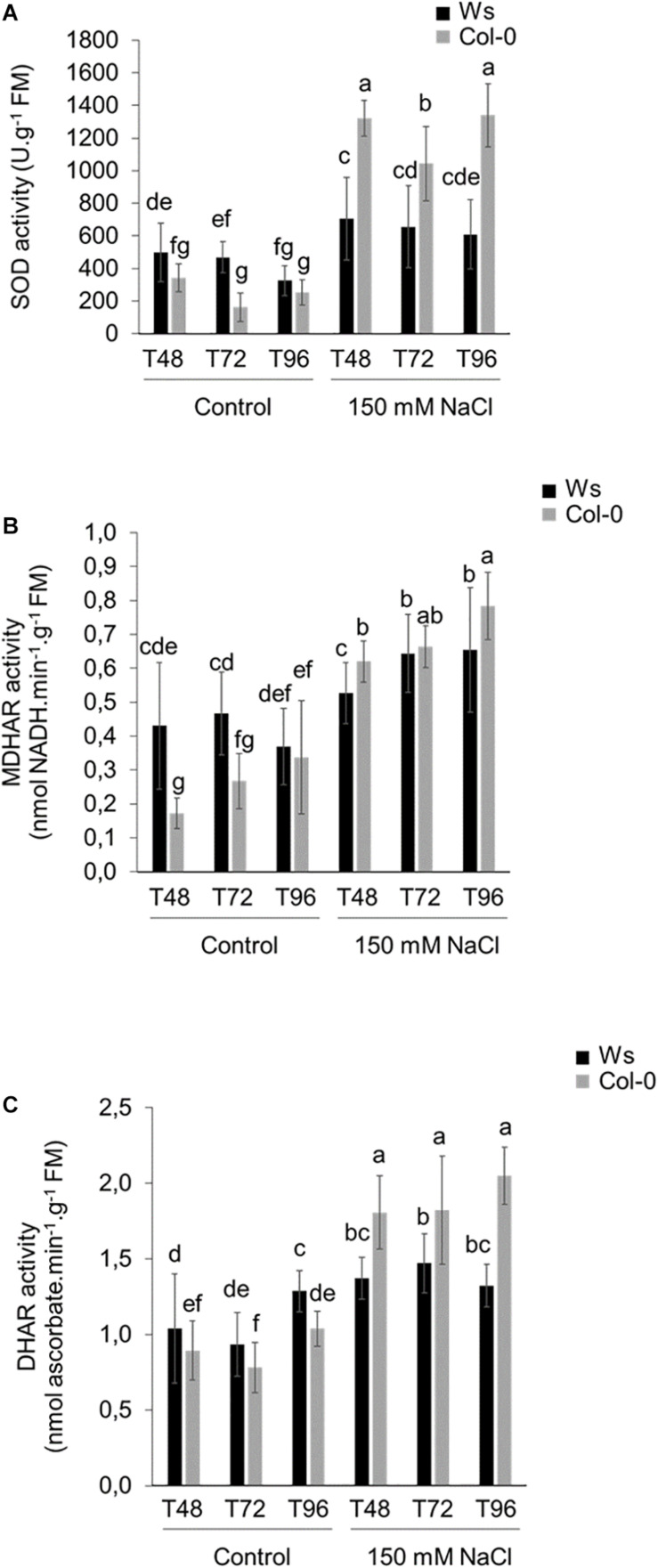
Antioxidant enzymatic activities in 3-week-old salt-treated Ws and Col-0 shoots. **(A)** SOD, **(B)** MDHAR, and **(C)** DHAR enzymatic activities measured under control conditions and 150 mM NaCl at T48, T72, and T96. Data are means ± standard deviation of three biological replicates. Means followed by the same letter in the same graph are not significantly different according to Dunn test.

Under control conditions, SOD, MDHAR, and DHAR activities had average values from 1.2- to 1.9-fold higher in Ws than in Col-0 (Figures 3A–C), whereas APOX, GR, and CAT activity levels were similar in both ecotypes (Supplementary Figures 3A–C). These ROS-scavenging enzymatic activities significantly increased in response to salt stress in both ecotypes. However, unlike control conditions, most antioxidant enzymatic activities were significantly higher in Col-0 as compared to Ws. SOD activity strongly increased by 4- to 6.5-fold in Col-0, whereas it was only 1.5- to 2-fold greater in Ws as compared to control plants (Figure 3A). MDHAR enzymatic activity increased more in Col-0 (2.8-fold) than in Ws (1.5-fold) to reach a similar activity level in both ecotypes (Figure 3B). DHAR enzymatic activity was at least twofold higher in salt-treated Col-0 shoots during the time course of the experiment as compared to Ws where a 1.3-fold increased level was observed (Figure 3C). CAT enzymatic activity was induced by more than twofold in both ecotypes, and APOX and GR enzymatic activities increased similarly in both ecotypes at T96 (Supplementary Figure 3).

### Primary Metabolic Changes in Col-0 and Ws Shoots Under Salt Stress

**FIGURE 4:**
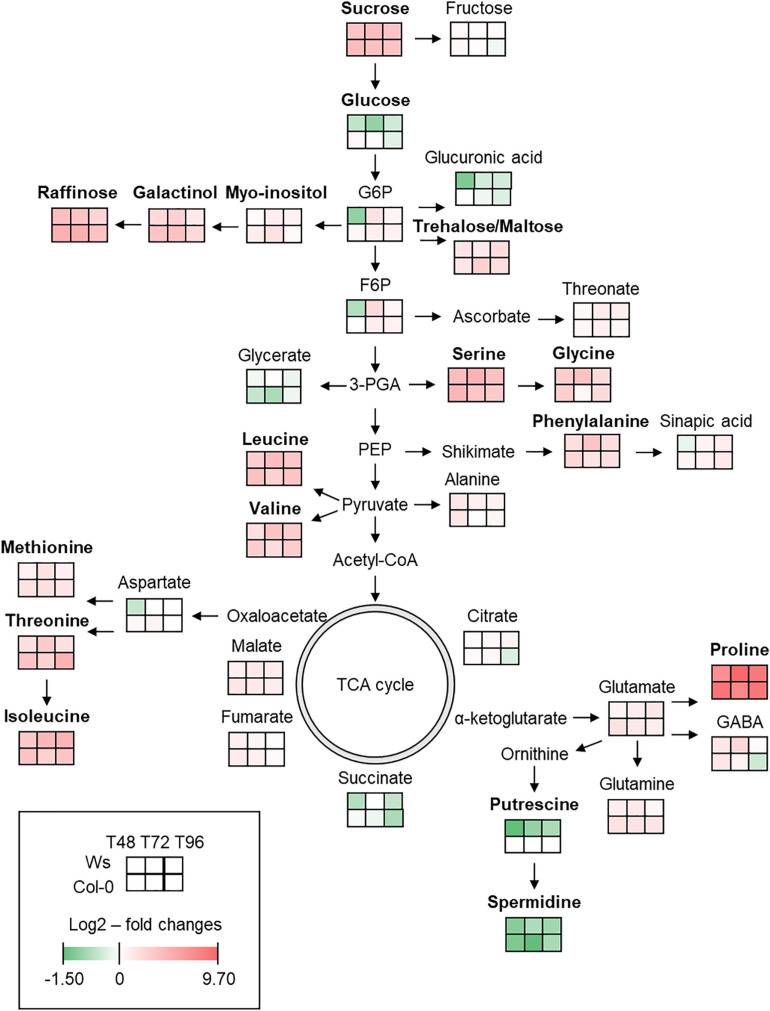
Primary metabolic map reflecting the metabolite changes in shoot of *Arabidopsis* Ws and Col-0 in response to salt stress at T48, T72, and T96. Means (*n* = 3) of log2–fold changes in metabolites content are represented as heatmap squares. The complete primary metabolites profile including statistical analysis can be found in Supplementary Table 1. Green and red colors represent, respectively, a lower and a higher metabolite content in the stress condition. In bold are the most discriminant metabolites.

To monitor the metabolic variations in both ecotypes under salt stress, metabolomics analysis was performed to identify differentially accumulated organic polar metabolites. In total, 33 primary metabolites (14 amino acids, 2 polyamines, 11 soluble sugars, and 6 organic acids) were reproducibly identified in the shoots of Ws and Col-0 in both control and salt-treated plants. The results are presented as the log2(salt/control)-fold change and integrated into a metabolites map (Figure 4 and Supplementary Table 1) as described by Wu et al. (2013).

Most primary metabolites were significantly affected upon salt stress exposure. Nitrogen metabolism was altered with a higher content of amino acids. Pro, which is the most frequently encountered compatible solute during an osmotic stress, was the most accumulated metabolite with an average value of nearly 8.5-fold in both ecotypes upon salt stress exposure. Contents of other amino acids, such as serine, leucine, isoleucine, and valine, increased by an average value of three- to fourfold in both ecotypes. Phenylalanine, derived from the shikimate pathway, increased greater in Ws (2.7-fold) than in Col-0 (2.2-fold) under salt stress. However, the highest accumulation of Pro, methionine, threonine, valine, glutamate, and glutamine occurred earlier in Col-0 (T48) as compared to Ws (T72 and T96). The level of non-protein amino acid, γ-aminobutyric acid, increased similarly at T48 in both ecotypes and continued to accumulate by 2.6-fold in Ws at T72 as compared to Col-0 where it started to decrease. The content in polyamines including putrescine and spermidine decreased in both ecotypes.

The carbohydrate metabolism also changed upon salt stress in both ecotypes as shown by an alteration of sugar content. Sucrose, a well-known osmolyte, was accumulated by an average value of fourfold in salt-treated plants. The amount of trehalose and maltose increased by more than twofold at T96 in both ecotypes, indicating a role of these disaccharides as a compatible solute and a starch remobilization under salt exposure. Raffinose, which is derived from the raffinose family of oligosaccharides (RFOs) biosynthesis pathway using myo-inositol and galactinol, was the most accumulated sugar in Col-0 with an average value of nearly fivefold vs. 3.7-fold in Ws upon salt treatment. Galactinol content increased in both ecotypes and was 1.5-fold greater in Col-0 than in Ws salt-treated plants.

### Sugar Composition of Non-cellulosic Cell Wall Polysaccharides in Ws and Col-0 Salt-Treated Shoots

As carbohydrate metabolism was altered in both Col-0 and Ws under salinity, the sugar composition of non-cellulosic cell wall polysaccharides was determined (Table 2 and Supplementary Table 2). Under control conditions, Ws contained more mannose (D-Man, 1.4-fold) and xylose (Xyl, 1.8-fold) residues than Col-0. Galactose (Gal) and galacturonic acid (GalUA) content was 1.3-fold higher in Col-0 than in Ws. Salt stress induced cell wall remodeling in both ecotypes. Pectin was altered in Col-0 as shown by a transient reduction by 1.4-fold in GalUA residues amount at T72. Additionally, salt treatment increased Gal content in Ws and Col-0. That was greater in Col-0 (1.5-fold) than in Ws (1.3-fold) at the end of the treatment, and changes occurred earlier in Col-0 as compared to Ws. Similarly, Ara content increased by 1.3-fold in both ecotypes, but that occurred earlier in Col-0 (T72) than in Ws (T96).

**TABLE 2:** Monosaccharide distribution of non-cellulosic cell wall polysaccharides in Ws and Col-0 at T48, T72, and T96 under control and 150 mM of NaCl conditions. l-Arabinose (Ara), d-galactose (Gal), d-xylose (Xyl), and l-galacturonic acid (GalUA) content.

**Mole%**		**Rha**	**Ara**	**Gal**	**Xyl**	**GalUA**
Ws control	T48	7.7 ± 0.2^ab^	12.2 ± 1.5^cd^	14.0 ± 0.4^d^	19.9 ± 5.9^bc^	34.3 ± 8.1^abc^
	T72	8.4 ± 0.1^a^	12.1 ± 1.8^cd^	13.7 ± 1.7^d^	22.9 ± 1.7^ab^	31.3 ± 2.5^bc^
	T96	7.2 ± 0.1^b^	12.1 ± 0.6^cd^	13.0 ± 0.0^d^	27.2 ± 2.0^a^	26.4 ± 2.5^cd^
Ws 150 mM NaCl	T48	7.4 ± 0.1^ab^	11.3 ± 0.4^d^	16.0 ± 0.7^c^	20.5 ± 0.0^bc^	34.1 ± 0.4^abc^
	T72	8.1 ± 0.7^ab^	12.8 ± 0.2^cd^	16.9 ± 3.5^c^	22.4 ± 7.6^ab^	29.6 ± 4.2^cd^
	T96	8.0 ± 0.8^ab^	15.3 ± 1.8^ab^	17.0 ± 1.5^c^	22.7 ± 1.8^ab^	23.3 ± 2.9^d^
Col-0 control	T48	8.7 ± 0.4^ab^	12.5 ± 0.8^cd^	17.6 ± 0.2^bc^	13.5 ± 4.4^*de*^	37.0 ± 3.4^ab^
	T72	8.4 ± 1.0^ab^	11.7 ± 2.2^cd^	16.3 ± 2.4^c^	13.2 ± 0.6^*de*^	40.0 ± 7.3^a^
	T96	8.0 ± 1.0^ab^	13.3 ± 0.8^c^	18.3 ± 2.6^bc^	15.3 ± 1.2^cd^	34.7 ± 2.1^abc^
Col-0 150 mM NaCl	T48	7.6 ± 0.0^a^	13.7 ± 0.0^bc^	20.4 ± 0.3^ab^	12.9 ± 0.1^e^	34.7 ± 0.2^ab^
	T72	7.3 ± 0.4^ab^	15.9 ± 1.4^ab^	24.0 ± 2.7^a^	13.2 ± 1.5^*de*^	28.9 ± 5.0^cd^
	T96	7.8 ± 1.1^ab^	17.9 ± 2.5^a^	20.1 ± 0.5^ab^	13.4 ± 1.5^*de*^	30.6 ± 4.6^bc^

*The complete non-cellulosic sugar composition profile can be found in Supplementary Table 2. Individual sugars are expressed in mole% of the total non-cellulosic cell wall polysaccharides. Data are means ± standard deviation (SD) of three biological replicates. Means followed by the same letter in the same series of values are not significantly different according to Dunn test.*

### Quantitative Proteomics Data Related to the Studied Traits in Col-0 and Ws Shoots

**FIGURE 5:**
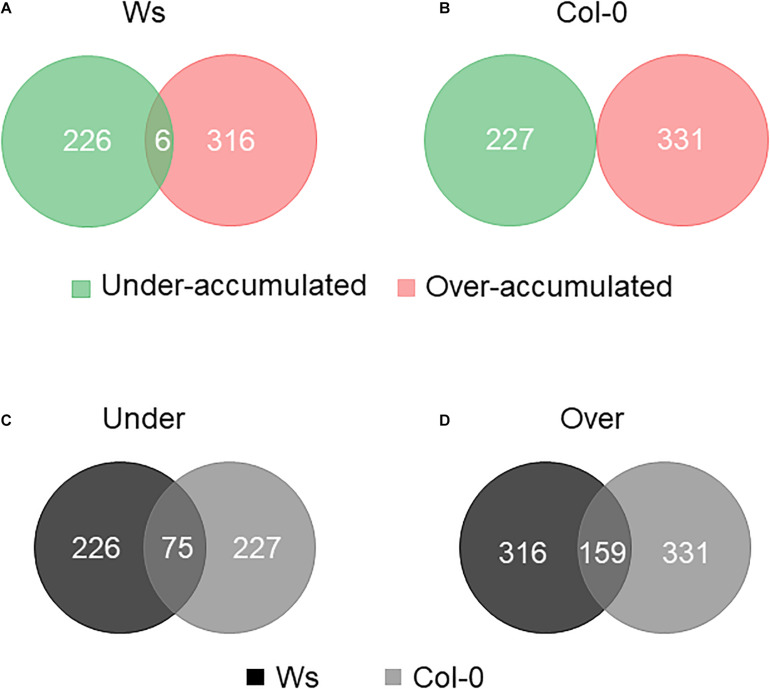
Venn diagram of differentially accumulated proteins (DAPs) in salt-treated Ws **(A)** and Col-0 **(B)** shoots. Number of common underaccumulated **(C)** and overaccumulated **(D)** proteins between Ws and Col-0 salt-treated shoots.

A TMTs-based comparative proteomic analysis was performed on total soluble proteins from the shoots of control and salt-treated plants. All the data described in this paragraph are available in Supplementary Tables 3, 4. Altogether, upon salt stress exposure, there were 542 DAPs in Ws and 558 in Col-0. The distribution of overaccumulated (316 Ws vs. 331 Col-0) and underaccumulated proteins (226 Ws vs. 227 Col-0) was similar in both ecotypes (Figures 5A,B). There were common 75 underaccumulated and 159 overaccumulated proteins between Ws and Col-0 (Figures 5C,D). Based on our physiological and biochemical data, we focused the following analysis on DAPs involved in photosynthesis, primary and secondary metabolisms, ROS detoxification, and cell wall.

**FIGURE 6:**
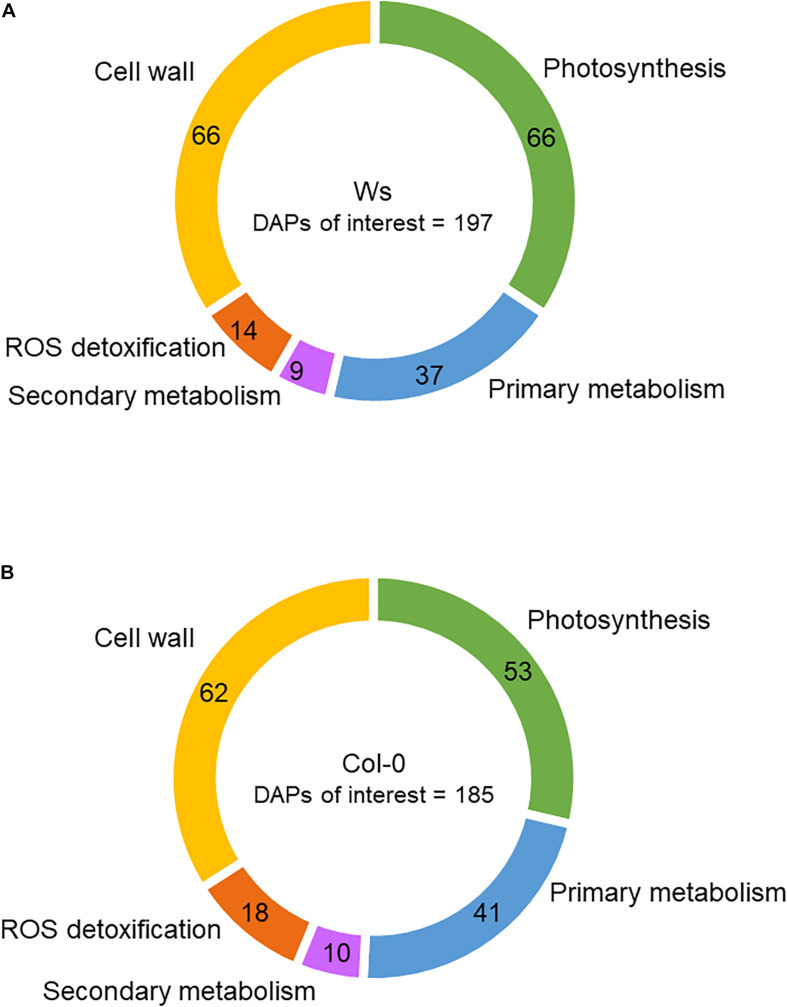
Functional categories of proteins differentially accumulated (DAPs) in the shoots of Ws **(A)** and Col-0 **(B)**. The numbers correspond to the distribution of DAPs among the different functional categories.

Among the DAPs, 53 (9.5%) and 66 (12%) were related to photosynthesis in Col-0 and Ws, respectively (Figure 6). The number of overaccumulated proteins was lower (13) than that of underaccumulated proteins (40) in Col-0, whereas in Ws, the numbers of proteins overaccumulated (28) and underaccumulated (32) were similar (Supplementary Tables 3, 4). Additionally, Ws contains six DAPs (petD, FD3, CURT1A and CURT1B, THF1, P19), which were either overaccumulated or underaccumulated, depending on the time points. The DAPs involved in photosynthetic pigment biosynthesis were more numerous in Col-0 (23) than in Ws (11). The amount of a pheophorbide a oxygenase (PaO) involved in chlorophyll catabolism nearly doubly increased at T96 in both Col-0 and Ws. Meanwhile, proteins involved in chlorophyll biosynthesis including chelatases (four in Col-0 vs. three in Ws), protochlorophyllide reductases (two in Col-0), and other chlorophyll biosynthesis–related proteins (nine in Col-0 vs. three in Ws) were underaccumulated upon salt stress treatment. The abundance of proteins related to carotenoid biosynthesis pathway as LUT2 and PSY1 was also reduced in both Col-0 and Ws. LUT5, and the carotenoid cleavage dioxygenase 4, CCD4, followed a similar trend (Supplementary Tables 5, 6).

The amount of proteins involved in starch catabolism (BAM5 and AMY1) strongly increased in Col-0 upon salt stress exposure (Supplementary Table 4). Other proteins related to starch degradation (SEX1/GWD1 and SEX4) were the most abundant in Ws at T96, whereas this occurred earlier in Col-0 (T48). Meanwhile, proteins involved in starch biosynthesis either overaccumulated (APL3, fourfold) in Col-0 and Ws shoots or underaccumulated (PTST3, twofold) in Col-0 at T96. Proteins related to starch/maltose to sucrose conversion (DPE2) or sucrose biosynthesis (SPS1) overaccumulated by 1.7-fold in Ws upon T72. The abundance of SUS1, which plays a role in directing the carbon flow to cell wall or starch biosynthesis, overdoubled in both Col-0 and Ws, but this occurred earlier in Col-0 (T48) than in Ws (T72). Sugar transport was also reduced in both ecotypes as shown with a lower abundance of SUC1 and STP1 transporters in Ws and Col-0, respectively.

Among the DAPs, 41 (7.4%) and 37 (6.8%) were related to primary metabolism in Col-0 and Ws, respectively (Figure 6). Raffinose synthase (RFS5) overaccumulated in Ws, whereas RFS6 was doubly reduced in both Ws and Col-0. RFS6 returned to its original level in Col-0 at T96, whereas it continued to decrease in Ws (Supplementary Tables 3, 4). The myo-inositol-1-phosphate synthase 1 (MIPS1) overaccumulated in both Col-0 and Ws, and that was twofold greater in Col-0 as compared to Ws at T48. Proteins involved in amino acid biosynthesis (ALS, VAT1, CGS1, LYSA1, CYSC1, HISN1A, SSU, ASN2, DHS2, and AK2) were underaccumulated in Ws and Col-0 shoots. The amount of delta-1-pyrroline-5-carboxylate synthase 1 (P5CS1) involved in Pro biosynthesis increased by twofold in both Ws and Col-0. Similarly, proteins involved in polyamine metabolism (ARGAH2 and DELTA-OAT) overaccumulated in both Ws and Col0. NATA1, which is involved in the reduction of putrescine level, strongly overaccumulated in Col-0 upon salt stress (more than sevenfold), whereas it was not detected in Ws.

Among the DAPs, 18 (3.2%) and 14 (2.6%) were related to ROS detoxification in Col-0 and Ws, respectively (Figure 6). CAT1, an SOD (MSD1), a glutathione peroxidase (GPX6), a DHAR (DHAR1), and several glutathione S-transferases (GSTF6, GSTF7, GSTF12, GSTL3, and GSTU16) were found in Ws and Col-0 (Supplementary Tables 3, 4). However, GSTs were more numerous in Col-0 (11) than in Ws (7). Additionally, MDAR3 overaccumulated only in Col-0, whereas DHAR2 specifically overaccumulated in Ws. Other GPXs overaccumulated either in Col-0 (GPX2) or in Ws (GPX1) as observed for APXs (APXS in Col0 vs. APX3 in Ws).

**FIGURE 7:**
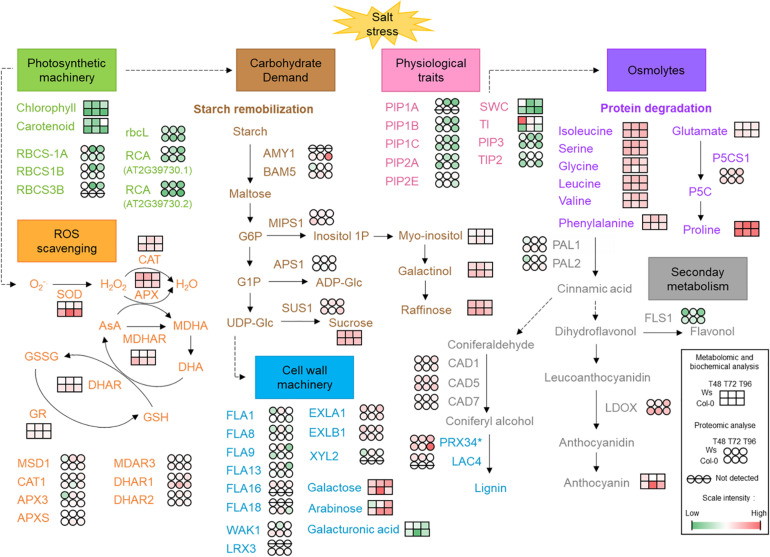
Physiological, biochemical, and molecular aspects identified in response to salt stress in two *Arabidopsis* ecotypes Ws and Col-0. AMY1, α-amylase 1; APS1, glucose-1-phosphate adenylyltransferase small subunit 1; APX, ascorbate peroxidase; AsA, ascorbic acid; BAM5, β-amylase 5; CAD, cinnamyl alcohol dehydrogenase; CAT, catalase; DHA, dehydroascorbate; DHAR, dehydroascorbate reductase; EXL, expansin-like; FLA, fascilin-like arabinogalactan protein; FLS1, flavonol synthase/flavanone 3-hydroxylase 1; G1P/G6P, glucose 1-phosphate/glucose 6-phosphate; GR, glutathione reductase; GSH; reduced glutathione; GSSG, glutathione-disulfide; Lac, laccase; LDOX, leucoanthocyanidin dioxygenase; LRX, leucine-rich repeat extension protein; MDAR3, monodehydroascorbate reductase 3; MDHA, monodehydroascorbate; MDHAR, monodehydroascorbate reductase; MIPS1, myo-inositol 1-phosphate synthase 1; MSD1, manganese superoxide dismutase 1; P5C, 1-pyrroline-5-carboxylate; P5CS1, delta-1-pyrroline-5-carboxylate synthase 1; PAL, phenylalanine ammonia lyase; PIP, plasma membrane intrinsic protein; Prx, cell wall peroxidase; RCA, ribulose bisphosphate carboxylase/oxygenase activase; rbcL, ribulose bisphosphate carboxylase large chain; RBCS, ribulose bisphosphate carboxylase small chain; ROS, reactive oxygen species; SOD, superoxide dismutase; SUS1, sucrose synthase 1; TI, shoot tolerance index; TIP, tonoplast intrinsic protein; SWC, shoot water content; UDP-Glc, uridine diphosphate glucose; WAK1, wall-associated kinase 1. *Prx34 may be involved in either lignin biosynthesis or ROS detoxification. In red, increased level/activity; in green, decreased level/activity; in white, steady-state level.

Secondary metabolism was also modulated in response to salt stress as shown by 9 (Col-0) and 10 (Ws) DAPs (Figure 7). Among DAPs, proteins involved in anthocyanin biosynthesis (LDOX and UGT75C1) overaccumulated in Col-0 and Ws (Supplementary Tables 3, 4). Proteins related to the early steps of the phenylpropanoid biosynthesis pathway (CHS, CHI, and F3H) were only overaccumulated in Col-0. Additionally, two phenylalanine ammonia lyases (PAL1 and PAL2) overaccumulated in Col-0.

Proteins predicted to be secreted were well represented among the DAPs. They were 62 and 66 (i.e., approximately 12%) in Col-0 and Ws, respectively, with 90% of them already identified in at least one cell wall proteome (see *WallProtDB*^4^). Twenty-three DAPs were common to Ws and Col-0, whereas 43 were specific to Ws and 39 to Col-0 (Supplementary Tables 3, 4). These proteins belonged to the main functional classes of cell wall proteins previously defined (Jamet et al., 2008). However, Ws and Col-0 exhibited distinct patterns: (i) there were more DAPs acting on cell wall polysaccharides (16 vs. 11), more miscellaneous proteins (10 vs. 6), and proteins of yet unknown function (9 vs. 5) in Col-0 than in Ws; and (ii) there were more DAPs related to lipid metabolism (11 vs. 3), more proteins with interaction domains with proteins or polysaccharides (11 vs. 6), and more proteins possibly involved in signaling (5 vs. 2) in Ws than in Col-0. Some protein families deserve additional comments. Among the DAPs acting on cell wall polysaccharides, four glycoside hydrolase (GH) families were specifically represented in Col-0: GH9 (endoglucanases), GH16 (xyloglucan endo-transglycosylases/hydrolases, XTH), GH32 (cell wall invertases), and GH35 (β-galactosidases). Besides, GH17 (endo-1,3-β-glucosidases) were more represented among Ws DAPs. GH19 (chitinases/lysozymes) and expansins were equally represented in Col-0 and Ws. Among the proteins possibly related to lipid metabolism, non-specific lipid transfer proteins and lipase acylesterases of the GDSL family were more represented among Ws DAPs, as well as lectins and fasciclin-like arabinogalactan proteins (FLAs). FLAs were highly represented in Ws (FLA1, FLA8, FLA9, FLA13, and FLA16) as compared to Col-0 (FLA18). One GH3 (BXL1, β-xylosidase 1) and two GH16 proteins (XTH4 and XTH6) underaccumulated by nearly twofold, whereas XTH24 overaccumulated by 1.8-fold in Col-0. A transient overaccumulation of one GH31 protein (Xyl2, a-xylanase 2) was observed at T72 in Ws. The amount of one GH35 protein (BGAL1, β-galactosidase 1) was almost halved at T48 and T72 in Col-0. The amount of one GH17 protein (BG2/PR2) was dramatically increased in Col-0 and Ws upon salt stress (24.5- and 8.8-fold at T96, respectively). Besides, a class III peroxidase (Prx34) was overaccumulated 7.6- and 3.8-fold at T96 in Col-0 and Ws, respectively.

## Discussion

In this study, two *Arabidopsis* accessions, Ws and Col-0, were used to identify new traits that could discriminate the degree of salt tolerance between them. We have evaluated the growth; the mineral status; photosynthetic pigments; antioxidative defense systems, such as ROS-scavenging enzymes, anthocyanin, and osmolytes from primary metabolism; and cell wall changes. We have combined physiological approaches with proteomics and metabolomics analyses (Figure 7).

Salinity tolerance is related to the ability to preserve steady water content and an accumulation of dry biomass as reported by Ellouzi et al. (2011). We observed a growth reduction in both ecotypes after salt exposure (Supplementary Figure 1A). That was correlated with lower SWC and TI values (Figure 1B and Supplementary Figure 1B). Aquaporins (PIPs and TIPs) play a key role in plant water relation and are involved in osmotic balance and nutrient homeostasis in response to salinity (Boursiac et al., 2005; Afzal et al., 2016; McGaughey et al., 2018). It has also been shown that PIPs could transport CO_2_ under water limiting conditions for photosynthesis capacity (Uehlein et al., 2012; Boudichevskaia et al., 2015). Our proteomics data were consistent with the water status of the shoots, which was reduced similarly in Ws and Col-0 in response to salt stress. We could identify two aquaporins (TIP2 and PIP1A) in Ws and one (PIP1D) in Col-0 whose amounts were salt-reduced by twofold (Supplementary Tables 5, 6). Therefore, according to McGaughey et al. (2018), the lower SWC and aquaporin abundance observed in both ecotypes suggest a limited capacity of salt-treated Ws and Col-0 plants to withstand from salt during a short period of stress. The steady water content under salt stress is related to the ability to exclude sodium from the leaves or ion compartmentalization capacity (Almeida et al., 2017). Both Ws and Col-0 showed a strong accumulation of Na^+^ ions in leaves, indicating an alteration of sodium homeostasis in plants (Table 1). We did not identify any Na^+^ transporter as HKT1 nor SOS proteins in our proteomics study. That can be explained by our extraction method, which mainly extracts soluble proteins, among many abundant photosynthesis-related proteins overlaying less abundant proteins during MS analysis. Under salt stress, K^+^ uptake is limited by the competition between Na^+^ influx and K^+^ efflux, which is stimulated by Na^+^ accumulation into the cell (Assaha et al., 2017). In our study, the excess of Na^+^ prevents K^+^ uptake in both Col-0 and Ws (Table 1). Many studies have reported that maintaining cellular Na^+^/K^+^ homeostasis is a key factor for salt tolerance (Mostofa et al., 2015; Singh et al., 2018). K^+^ is an essential macronutrient involved in not only photoprotection and optimal photosynthetic chain electron transport (Dukic et al., 2019) but also regulation of intracellular and chloroplast osmolarity (Barragán et al., 2012; Tsujii et al., 2019). The amount of K^+^/H^+^ antiporters (KEA-1, KEA-2, and KEA-3), which are located in chloroplast (Kunz et al., 2014) and may play an important role in the initial rapid Ca^2+^ response to hyperosmotic stress (Stephan et al., 2016; Tsujii et al., 2019), remained unchanged in Ws, whereas the amount of KEA1 and KEA3 likely reduced (0.7-fold) at T96 in Col-0 (Supplementary Tables 5, 6). Although we observed a similar Na^+^ accumulation in both ecotypes, it is possible that Col-0 limits excess of Na^+^ by closing the K^+^ channels KEA1 and KEA3. This indicates not only a stronger alteration of K^+^ transport activity, but also a limited photosynthetic capacity more pronounced in Col-0 than in Ws.

Photosynthetic pigments are altered under salt stress (Esteban et al., 2015; Ben Abdallah et al., 2016). The photosynthetic pigments were reduced in both ecotypes and were stronger in Col-0 than in Ws (Supplementary Figure 2). These data correlated well with the proteomics data where proteins involved in the PaO pathway (i.e., chlorophyll degradation) and carotenoids biosynthesis pathway overaccumulated and underaccumulated, respectively, greater in Col-0 than in Ws upon salt stress (Supplementary Tables 3, 4). Additionally, more proteins related to the photosynthetic electron transport chain are underaccumulated in Col-0, whereas most of them are overaccumulated in Ws. All these data indicate that Ws seems to maintain its photosynthetic capacity at a higher level than Col-0 under salinity.

To safeguard their photosystems under salt stress, but also to maintain cellular homeostasis or carbon storage, plants accumulate osmoprotectants as soluble sugars (Nishizawa et al., 2008; Schneider and Keller, 2009; Knaupp et al., 2011; ElSayed et al., 2014), anthocyanin (Kim et al., 2017), or amino acids (Dong et al., 2018). Starch remobilization is important to provide carbon and energy when photosynthesis is limited under abiotic stress (Thalmann and Santelia, 2017). It provides organic acids, sugars, and sugar-derived osmolytes for osmotic adjustment (Monroe et al., 2014; Santelia and Lunn, 2017). Thalmann et al. (2016) showed that the reduction in starch accumulation in *Arabidopsis* plants exposed to high osmotic stress results from induced starch degradation, leading to the production of maltose, the major starch catabolite. We did not measure the starch content, but the level of maltose increased in both ecotypes and was twofold greater in Col-0 than in Ws at T72 (Supplementary Table 1). That was consistent with an overaccumulation of proteins involved in starch breakdown as AMY1, BAM5 (Col-0), GWD1 (Ws), and SEX4 (Col-0 and Ws) (Supplementary Tables 3, 4). Interestingly, APL3 (AGPase), a key enzyme of starch biosynthesis pathway, overaccumulated in both ecotypes and to a higher level in Col-0. This indicates that salt stress stimulates both starch catabolism and biosynthesis in Ws and Col-0 to mitigate salt stress (Kempa et al., 2008; Thalmann and Santelia, 2017). Sucrose increased by fourfold in both ecotypes under salinity. That was consistent with the abundance of the proteins SPS1 and DPE2 in Ws. SUS1 promotes sucrose degradation and has been shown to be possibly involved in cellulose and starch biosynthesis in *Arabidopsis* (Baroja-Fernández et al., 2012). SUS1 overaccumulated in both Col-0 and Ws, but that was greater and earlier in Col-0 than in Ws. Furthermore, the decreased amount of a cell wall invertase (cwINV1), a sugar transport protein (STP1) in Col-0, and a sucrose transport protein (SUC1) in Ws suggests a reduction in carbon partitioning with consequences on source–sink relationships (Thalmann et al., 2016).

Sugar osmolytes such as raffinose or galactinol are derivatives of sucrose and were more abundant in Col-0 than in Ws (Figure 4 and Supplementary Table 1). Surprisingly, we observed a strong underaccumulation of the raffinose synthase RFS5, in both Col-0 and Ws. However, the level of RFS5 was recovered in Col-0 at T96, and the amount of another raffinose synthase, RFS6, doubly increased in Ws upon salt stress. Raffinose biosynthesis could also arise from stachyose catabolism through raffinose a-galactosidase (AGAL) activity. We identified two AGALs (AGAL1 and AGAL2) in both ecotypes where AGAL1 increased by 1.6-fold upon T72 in Col-0, but not in Ws (Supplementary Tables 5, 6). The higher levels of raffinose and galactinol measured in Col-0 may be important as osmoprotectants to reduce the oxidative damages.

Pro, another compatible solute, as well as other amino acids (serine, glycine, phenylalanine, valine, threonine, leucine, and isoleucine), accumulated in both ecotypes (Figure 4 and Supplementary Table 1). However, Pro, valine, leucine, and phenylalanine levels were higher in Col-0 at T48. Additionally, P5CS1, which contributes to stress-induced Pro accumulation, was twice more abundant under salt stress in both ecotypes, which agrees with a former study on *atp5cs* mutants exposed to salt stress (Funck et al., 2020). Underaccumulated proteins involved in amino acid biosynthesis were more abundant in Col-0 than in Ws, and the amount of overaccumulated proteins involved in amino acid catabolism was similar in both ecotypes. This suggests an active protein degradation in response to salt stress in both ecotypes, and these changes often occurred earlier in Col-0 than in Ws. Increased amounts of these amino acids can be considered as a salt tolerance mechanism to promote osmolytes or nitrogen allocation to produce non-protein amino acids or secondary metabolites (Kempa et al., 2008; Dong et al., 2018; Batista-Silva et al., 2019; Jia et al., 2019). Their complete oxidation could also provide a source of energy possibly required to prepare the recovery phase after salt stress (Hildebrandt et al., 2015).

Anthocyanin acts as an antioxidant molecule and is actively synthesized under salt stress (Chutipaijit et al., 2011; Zhang et al., 2012; Cheng et al., 2013; Landi et al., 2015; Kim et al., 2017). The overaccumulation of anthocyanin in response to stress reduces photosynthesis efficiency (Chalker-Scott, 1999; Gould et al., 2000). In our study, the anthocyanin content increased greater in Col-0 than in Ws (Figure 2). These results are supported by the work of Chunthaburee et al. (2016), which showed that salt-tolerant rice cultivars displayed smaller increase in anthocyanin content than salt-sensitive cultivars. Specific proteins involved in anthocyanin biosynthesis such as LDOX and one glycosyltransferase (UGT75C1) were more abundant in Col-0 than in Ws (Supplementary Tables 3, 4). Furthermore, the amount of proteins involved in the early steps of the phenylpropanoid pathway (F3H, CHS, and CHI) increased in Col-0. Therefore, the overaccumulation of anthocyanin observed in Col-0 likely appears more as a trait of sensitivity to salt tolerance.

Other mechanisms, such as ROS-scavenging enzymes, including SOD, CAT, and APOX, have been developed in plants to alleviate the oxidative damages caused by ROS (Mittler, 2002; Ahanger et al., 2017; Gupta et al., 2018). In our study, an increase in antioxidative enzymatic activities was observed in both ecotypes in response to salt stress (Figure 3 and Supplementary Figure 3). Most of these activities were higher in Col-0 than in Ws (Figure 3). The lower increase in antioxidative enzymatic activity observed in Ws under salt stress could be explained by a greater activity under control conditions. SOD enzymatic activity, which acts as the first line of defense against oxidative damage in chloroplasts, was higher in Col-0 than in Ws, suggesting that a stronger oxidative stress was generated in Col-0. Surprisingly, the amount of the SOD MSD1 protein was greater in Ws in response to salt stress (Supplementary Tables 3, 4). However, other SODs (FSD1, FSD2, FSD3, CSD1, and CSD2) were identified in both ecotypes, and the abundance of FSD3, CSD1, and 2 likely decreased in Ws, whereas they remained unchanged or slightly increased (CSD2) in Col-0, indicating a higher H_2_O_2_ production in Col-0 (Supplementary Tables 5, 6). The correlation observed between SOD enzymatic activities and SOD protein abundance was also shown for the enzymes involved in H_2_O_2_ detoxification. The detoxification of H_2_O_2_ was favored in Col-0 by the ascorbate–glutathione cycle, whereas Ws promotes CAT activity. Therefore, the oxidative metabolism seems to be better regulated in Ws than in Col-0, with coordinated SOD and CAT activities allowing to keep a balance between ROS production and detoxification and to reduce oxidative load to ascorbate–glutathione cycle, while GR activity maintains the redox status (Mostofa et al., 2015).

The cell wall is the first interface between the plant cell and its environment and has important role in the perception of environmental stimuli, resulting in activation of cellular responses to biotic and abiotic stresses (Le Gall et al., 2015; Feng et al., 2018; Novaković et al., 2018; Zhao et al., 2018). We observed a transient reduction in pectin in Col-0 and an increase of arabinan and galactan in both ecotypes after salt exposure. Xyl content remained unchanged in both ecotypes under salt stress (Table 2). That can be explained by the decreased abundance of a bifunctional β-L-arabinofuranosidase/β-D-xylosidase (BXL1) in Col-0 (Supplementary Table 4). A similar trend occurred in Ws (Supplementary Table 5). In *Arabidopsis* vascular tissues, BXL1 releases Xyl from xylan and arabinoxylan, but also arabinose from arabinoxylan and arabinan (Minic et al., 2004). The decrease in BXL1 abundance observed in Col-0 and to a certain extent in Ws could maintain arabinoxylan and arabinan content in our conditions. Additionally, a decrease in XTH4 and XTH6 (T48 and T72) abundance in Col-0 concomitantly to an increase in XTH24 (T96) could contribute to maintain the xyloglucan (XG) level in Col-0 (Supplementary Table 4). Besides, salt stress transiently reduced the amount of GalUA residues in Col-0 (T72), indicating pectin degradation (Table 2). Pectin is essential for the CWI (Bethke et al., 2016; Feng et al., 2018). Feng et al. (2018) reports that the FER is a sensor of the CWI, which interacts with the salt-induced disruption of pectin and induces cell wall repair by triggering a cytoplasmic Ca^2+^ signaling pathway. Zhao et al. (2018) showed that LRX3/4/5, RALF22/23, and FER are critical to maintain CWI under salt stress. We identified LRX3 in Col-0 but not in Ws, and the amount likely increased in response to salt stress in Col-0 (Supplementary Table 6). FER, rapid alkalinization factors (RALFs), and other LRXs, which are crucial players of the signaling pathway regulating plant growth under salt stress, might be synthesized earlier than at T48 in salt-treated plants. We could also identify a cell wall–associated kinase WAK1 in Col-0, which can activate plant stress response after binding to pectin fragments (Brutus et al., 2010; Kohorn and Kohorn, 2012). The abundance of WAK1 doubly increased in Col-0 up to T72 (Supplementary Table 4). Thus, the reduction in GalUA residues concomitantly with the increase in WAK1 abundance observed in Col-0 suggests the existence of a cell wall signal to maintain CWI in Col-0. Arabinan and galactan increased in both ecotypes, but started earlier in Col-0. These polysaccharides are mainly found in rhamnogalacturonan I (RG-I) side chains, which play a role in the hydration status of the cell wall matrix for their high water-binding capability and ability to form gels that could preserve cells from damage (Leucci et al., 2008). Zhao et al. (2019) reported that Ara is important to maintain root growth under salt stress in *Arabidopsis*. Ara and Gal residues also decorate hydroxyproline-rich glycoproteins such as arabinogalactan proteins (AGPs) and extensins (Draeger et al., 2015). Olmos et al. (2017) showed that AGPs are released under salt stress to maintain CWI and suggests a role of AGP in Na^+^/Ca^2+^ exchange. The increased amounts of Ara and Gal residues observed in both genotypes suggest rather an increase in AGPs to maintain CWI under salt stress. AtFLA4/SOS5, which is a fascilin-like AGP, positively regulates cell wall biosynthesis and root growth by modulating ABA signaling in response to salt stress (Seifert et al., 2014). Shi et al. (2003) have proposed that AtFLA4 is involved in ionic interactions, most likely with other FLAs, such that they form a network at either the plasma membrane surface, or in the cell wall that controls the rate of cell expansion. We identified one FLA (FLA18) in Col-0 whose amount doubled at T72 and five different FLAs (FLA1, FLA8, FLA9, FLA13, and FLA16) in Ws whose amounts nearly tripled at T72. Besides FLA16, other FLAs overaccumulated in Ws and could be detected in Col-0 but with no change. FLA16 has been shown to be involved in stem development (Liu et al., 2020) and was not identified in Col-0, which could be explained by its rosette leaves stage. Thus, the overaccumulated FLAs observed in Ws, except for FLA16, could be important in the maintenance of CWI. Similarly, FLA18, WAK1, and LRX3, which have been found in Col-0, could be good candidates for the maintenance of CWI in salt tolerance. Expansin is able to promote cell wall expansion under a low apoplastic pH (Cosgrove, 2000). AtEXLA1 and AtEXLB1 overaccumulated in both Ws and Col-0, but the abundance was higher in Ws than in Col-0 at T48. RALFs induced a rapid alkalinization of the apoplast upon salt stress (Zhao et al., 2019). Thus, the overaccumulation of expansins under salt stress would not favor cell wall expansion but rather participate to the salt stress signaling pathway. Several studies showed that overexpression of an expansin gene in *Arabidopsis* was shown to confer enhanced salt tolerance by lowering ROS accumulation and increasing antioxidant activity (Abuqamar et al., 2013; Lü et al., 2013; Chen et al., 2019; Jadamba et al., 2020). Thus, the higher abundance of FLAs associated with a higher overaccumulation of expansins observed in Ws could contribute to a better CWI in response to salt stress than Col-0, where only one FLA and WAK1 were overaccumulated. Most of these experiments showed a transient change at T72 in both ecotypes. The T72 kinetic point more than likely corresponds to a critical metabolism change point, which could determine subsequent salt adaptation or cell death.

## Conclusion

In conclusion, our results showed that salt stress alters several metabolic pathways in *Arabidopsis*. Both ecotypes adopt a similar global strategy to cope with salt stress. However, Ws is less salt-sensitive than Col-0, and the degree of salt tolerance could be rather related to the stage of development than to their respective genetic background.

## Data Availability Statement

The MS/MS data (raw data, identification, and quantification results) are available on ProteomeXchange (http://www.proteomexchange.org) with identifier PXD022441. The data are publicly available and have been published (Leschevin et al., 2021: https://doi.org/10.1002/pmic.202000293).

## Author Contributions

CR, KP, and ML designed the experiments. ML performed most of the experiments. SB helped for hydroponics culture. MI performed anthocyanin content and helped to determine antioxidant enzymes activities. AQ carried out the metabolites and ions analysis with the help of ML. CR and RR analyzed cell wall sugar composition. PM, KP, and ML performed proteomics analysis. EJ and HS analyzed and organized the proteomics data. ML, MI, EJ, and CR wrote the manuscript. All authors read and approved the manuscript.

## Conflict of Interest

The authors declare that the research was conducted in the absence of any commercial or financial relationships that could be construed as a potential conflict of interest.

## Footnotes

1 https://www.expasy.org/resources/uniprotkb-swiss-prot

2 https://www.uniprot.org

3 http://www.proteomexchange.org

4 http://www.polebio.lrsv.ups-tlse.fr/WallProtDB

## Supplementary Material

Supplementary Figure 1 Shoot biomass and shoot tolerance index.

Supplementary Figure 2 Photosynthetic pigment content (mg ⋅ g^–1^DM) in Ws and Col-0 control and salt-treated plants.

Supplementary Figure 3 Enzymatic activities of oxidoreductases in Ws and Col-0 control and salt-treated plants.

Supplementary Table 1 Quantification of amino acids, carbohydrates, and organic acids in Ws and Col-0 control and salt-treated plants.

Supplementary Table 2 Non-cellulosic monosaccharide composition of the cell wall (mole%) in Ws and Col-0 control and salt-treated plants.

Supplementary Table 3 Differentially accumulated proteins identified in shoots of Ws salt-treated plants vs. control plants with a fold change >1.7 or <0.6 at a *p* < 0.05.

Supplementary Table 4 Differentially accumulated proteins identified in shoots of Col-0 salt-treated plants vs. control plants with a fold change >1.7 or <0.6 at a *p* < 0.05.

Supplementary Table 5 Differentially accumulated proteins identified in shoots of Ws salt-treated plants vs. control plants at a *p* < 0.05.

Supplementary Table 6 Differentially accumulated proteins identified in Col-0 shoots of salt-treated plants vs. control plants at a *p* < 0.05.

## References

[B1] Abuqamar S. Ajeb S. Sham A. Enan M. R. Iratni R. (2013). A mutation in the *expansin-like A2* gene enhances resistance to necrotrophic fungi and hypersensitivity to abiotic stress in *Arabidopsis thaliana*. *Mol. Plant Pathol.* 14 813–827. 10.1111/mpp.12049 23782466 PMC6638991

[B2] Acosta-Motos J. R. Ortuño M. F. Bernal-Vicente A. Diaz-Vivancos P. Sanchez-Blanco M. J. Hernandez J. A. (2017). Plant responses to salt stress: adaptive mechanisms. *Agronomy* 7:18. 10.20944/preprints201702.0083.v2 32283112

[B3] Afzal Z. Howton T. C. Sun Y. Mukhtar M. S. (2016). The roles of aquaporins in plant stress responses. *J. Dev. Biol.* 4:9. 10.3390/jdb4010009 29615577 PMC5831814

[B4] Ahanger M. A. Tomar N. S. Tittal M. Argal S. Agarwal R. M. (2017). Plant growth under water/salt stress: ROS production; antioxidants and significance of added potassium under such conditions. *Physiol. Mol. Biol. Plants* 23 731–744. 10.1007/s12298-017-0462-7 29158624 PMC5671444

[B5] Almeida D. M. Oliveira M. M. Saibo N. J. M. Almeida D. M. Oliveira M. M. Saibo N. J. M. (2017). Regulation of Na^+^ and K^+^ homeostasis in plants: towards improved salt stress tolerance in crop plants. *Genet. Mol. Biol.* 40 326–345. 10.1590/1678-4685-gmb-2016-0106 28350038 PMC5452131

[B6] Apel K. Hirt H. (2004). Reactive oxygen species: metabolism, oxidative stress, and signal transduction. *Annu. Rev. Plant Biol.* 55 373–399. 10.1146/annurev.arplant.55.031903.141701 15377225

[B7] Assaha D. V. M. Ueda A. Saneoka H. Al-Yahyai R. Yaish M. W. (2017). The role of Na^+^ and K^+^ transporters in salt stress adaptation in glycophytes. *Front. Physiol.* 8:509. 10.3389/fphys.2017.00509 28769821 PMC5513949

[B8] Baroja-Fernández E. Munoz F. J. Li J. Bahaji A. Almagro G. Montero M. (2012). Sucrose synthase activity in the sus1/sus2/sus3/sus4 *Arabidopsis* mutant is sufficient to support normal cellulose and starch production. *Proc. Natl. Acad. Sci. U.S.A.* 109 321–326. 10.1073/pnas.1117099109 22184213 PMC3252950

[B9] Barragán V. Leidi E. O. Andrés Z. Rubio L. De Luca A. Fernández J. A. (2012). Ion exchangers NHX1 and NHX2 mediate active potassium uptake into vacuoles to regulate cell turgor and stomatal function in *Arabidopsis*. *Plant Cell* 24 1127–1142. 10.1105/tpc.111.095273 22438021 PMC3336136

[B10] Bassil E. Ohto M. Esumi T. Tajima H. Zhu Z. Cagnac O. (2011). The *Arabidopsis* intracellular Na^+^/H^+^ antiporters NHX5 and NHX6 are endosome associated and necessary for plant growth and development. *Plant Cell* 23 224–239. 10.1105/tpc.110.079426 21278129 PMC3051250

[B11] Batista-Silva W. Heinemann B. Rugen N. Nunes-Nesi A. Araújo W. L. Braun H.-P. (2019). The role of amino acid metabolism during abiotic stress release. *Plant Cell Environ.* 42 1630–1644. 10.1111/pce.13518 30632176

[B12] Baxter I. Brazelton J. N. Yu D. Huang Y. S. Lahner B. Yakubova E. (2010). A coastal cline in sodium accumulation in *Arabidopsis thaliana* is driven by natural variation of the sodium transporter AtHKT1;1. *PLoS Genet.* 6:e1001193. 10.1371/journal.pgen.1001193 21085628 PMC2978683

[B13] Ben Abdallah S. Aung B. Amyot L. Lalin I. Lachâal M. Bouraoui-Karray N. (2016). Salt stress (NaCl) affects plant growth and branch pathways of carotenoid and flavonoid biosyntheses in *Solanum nigrum*. *Acta Physiol. Plant.* 38:72. 10.1007/s11738-016-2096-8

[B14] Berthomieu P. Conéjéro G. Nublat A. Brackenbury W. J. Lambert C. Savio C. (2003). Functional analysis of *AtHKT1* in *Arabidopsis* shows that Na+ recirculation by the phloem is crucial for salt tolerance. *EMBO J.* 22 2004–2014. 10.1093/emboj/cdg207 12727868 PMC156079

[B15] Bethke G. Thao A. Xiong G. Li B. Soltis N. E. Hatsugai N. (2016). Pectin biosynthesis is critical for cell wall integrity and immunity in *Arabidopsis thaliana*. *Plant Cell* 28 537–556. 10.1105/tpc.15.00404 26813622 PMC4790862

[B16] Bose J. Rodrigo-Moreno A. Shabala S. (2014). ROS homeostasis in halophytes in the context of salinity stress tolerance. *J. Exp. Bot.* 65 1241–1257. 10.1093/jxb/ert430 24368505

[B17] Boudichevskaia A. Heckwolf M. Kaldenhoff R. (2015). T-DNA insertion in aquaporin gene *AtPIP1;2* generates transcription profiles reminiscent of a low CO_2_ response. *Plant Cell Environ.* 38 2286–2298. 10.1111/pce.12547 25850563

[B18] Boursiac Y. Chen S. Luu D.-T. Sorieul M. van den Dries N. Maurel C. (2005). Early effects of salinity on water transport in *Arabidopsis* roots. Molecular and cellular features of aquaporin expression. *Plant Physiol.* 139 790–805. 10.1104/pp.105.065029 16183846 PMC1255996

[B19] Brunetti C. Guidi L. Sebastiani F. Tattini M. (2015). Isoprenoids and phenylpropanoids are key components of the antioxidant defense system of plants facing severe excess light stress. *Environ. Exp. Bot.* 119 54–62. 10.1016/j.envexpbot.2015.04.007

[B20] Brutus A. Sicilia F. Macone A. Cervone F. Lorenzo G. D. (2010). A domain swap approach reveals a role of the plant wall-associated kinase 1 (WAK1) as a receptor of oligogalacturonides. *Proc. Natl. Acad. Sci. U.S.A.* 107 9452–9457. 10.1073/pnas.1000675107 20439716 PMC2889104

[B21] Carpita N. C. Gibeaut D. M. (1993). Structural models of primary cell walls in flowering plants: consistency of molecular structure with the physical properties of the walls during growth. *Plant J.* 3 1–30. 10.1111/j.1365-313X.1993.tb00007.x 8401598

[B22] Chalker-Scott L. (1999). Environmental significance of anthocyanins in plant stress responses. *Photochem. Photobiol.* 70 1–9. 10.1111/j.1751-1097.1999.tb01944.x

[B23] Chaves M. M. Flexas J. Pinheiro C. (2009). Photosynthesis under drought and salt stress: regulation mechanisms from whole plant to cell. *Ann. Bot.* 103 551–560. 10.1093/aob/mcn125 18662937 PMC2707345

[B24] Chen Y. Zhang B. Li C. Lei C. Kong C. Yang Y. (2019). A comprehensive expression analysis of the expansin gene family in potato (*Solanum tuberosum*) discloses stress-responsive expansin-like B genes for drought and heat tolerances. *PLoS One* 14:e0219837. 10.1371/journal.pone.0219837 31318935 PMC6638956

[B25] Cheng Y.-J. Kim M.-D. Deng X.-P. Kwak S.-S. Chen W. (2013). Enhanced salt stress tolerance in transgenic potato plants expressing IbMYB1, a sweet potato transcription factor. *J. Microbiol. Biotechnol.* 23 1737–1746. 10.4014/jmb.1307.07024 24378636

[B26] Chunthaburee S. Dongsansuk A. Sanitchon J. Pattanagul W. Theerakulpisut P. (2016). Physiological and biochemical parameters for evaluation and clustering of rice cultivars differing in salt tolerance at seedling stage. *Saudi J. Biol. Sci.* 23 467–477. 10.1016/j.sjbs.2015.05.013 27298579 PMC4890196

[B27] Chutipaijit S. Cha-Um S. Sompornpailin K. (2011). High contents of proline and anthocyanin increase protective response to salinity in *Oryza sativa* L. spp. indica. *Aust. J. Crop Sci.* 5 1191–1198.

[B28] Cosgrove D. J. (2000). Loosening of plant cell walls by expansins. *Nature* 407 321–326. 10.1038/35030000 11014181

[B29] Davenport R. J. Muñoz-Mayor A. Jha D. Essah P. A. Rus A. Tester M. (2007). The Na^+^ transporter AtHKT1;1 controls retrieval of Na^+^ from the xylem in *Arabidopsis*. *Plant Cell Environ.* 30 497–507. 10.1111/j.1365-3040.2007.01637.x 17324235

[B30] Dong S. Zhang J. Beckles D. M. (2018). A pivotal role for starch in the reconfiguration of 14 C-partitioning and allocation in *Arabidopsis thaliana* under short-term abiotic stress. *Sci. Rep.* 8:9314. 10.1038/s41598-018-27610-y 29915332 PMC6006365

[B31] Draeger C. Ndinyanka Fabrice T. Gineau E. Mouille G. Kuhn B. M. Moller I. (2015). *Arabidopsis* leucine-rich repeat extensin (LRX) proteins modify cell wall composition and influence plant growth. *BMC Plant Biol.* 15:155. 10.1186/s12870-015-0548-8 26099801 PMC4477543

[B32] Duan L. Dietrich D. Ng C. H. Chan P. M. Y. Bhalerao R. Bennett M. J. (2013). Endodermal ABA signaling promotes lateral root quiescence during salt stress in *Arabidopsis* seedlings. *Plant Cell* 25 324–341. 10.1105/tpc.112.107227 23341337 PMC3584545

[B33] Dukic E. Herdean A. Cheregi O. Sharma A. Nziengui H. Dmitruk D. (2019). K+ and Cl^–^ channels/transporters independently fine-tune photosynthesis in plants. *Sci. Rep.* 9:8639. 10.1038/s41598-019-44972-z 31201341 PMC6570773

[B34] Dunn O. J. (1964). Multiple comparisons using rank sums. *Technometrics* 6 241–252. 10.1080/00401706.1964.10490181

[B35] Duran Garzon C. Lequart M. Rautengarten C. Bassard S. Sellier-Richard H. Baldet P. (2020). Regulation of carbon metabolism in two maize sister lines contrasted for chilling tolerance. *J. Exp. Bot.* 71 356–369. 10.1093/jxb/erz421 31557299

[B36] Edelenbos M. Christensen L. P. Grevsen K. (2001). HPLC determination of chlorophyll and carotenoid pigments in processed green pea cultivars (*Pisum sativum* L.). *J. Agric. Food Chem.* 49 4768–4774. 10.1021/jf010569z 11600019

[B37] Ellouzi H. Hamed K. B. Cela J. Munné-Bosch S. Abdelly C. (2011). Early effects of salt stress on the physiological and oxidative status of *Cakile maritima* (halophyte) and *Arabidopsis thaliana* (glycophyte). *Physiol. Plant.* 142 128–143. 10.1111/j.1399-3054.2011.01450.x 21288246

[B38] ElSayed A. I. Rafudeen M. S. Golldack D. (2014). Physiological aspects of raffinose family oligosaccharides in plants: protection against abiotic stress. *Plant Biol.* 16 1–8. 10.1111/plb.12053 23937337

[B39] Eryılmaz F. (2006). The relationships between salt stress and anthocyanin content in higher plants. *Biotechnol. Biotechnol. Equip.* 20 47–52. 10.1080/13102818.2006.10817303

[B40] Esteban R. Barrutia O. Artetxe U. Fernandez-Marin B. Hernandez A. Garcia-Plazaola J. I. (2015). Internal and external factors affecting photosynthetic pigment composition in plants: a meta-analytical approach. *New Phytol.* 206 268–280. 10.1111/nph.13186 25414007

[B41] Ewing J. F. Janero D. R. (1995). Microplate superoxide dismutase assay employing a nonenzymatic superoxide generator. *Anal. Biochem.* 232 243–248. 10.1006/abio.1995.0014 8747482

[B42] FAO (2018). *Handbook for Saline Soil Management*, eds Vargas R. Pankova E. I. Balyuk S. A. Krasilnikov P. V. Khasankhanova G. M. (Rome: Food and Agriculture Organization of the United Nations).

[B43] Feng W. Kita D. Peaucelle A. Cartwright H. N. Doan V. Duan Q. (2018). The FERONIA receptor kinase maintains cell-wall integrity during salt stress through Ca^2+^ signaling. *Curr. Biol.* 28 666–675.e5. 10.1016/j.cub.2018.01.023 29456142 PMC5894116

[B44] Fleischer A. O’Neill M. A. Ehwald R. (1999). The pore size of non-graminaceous plant cell walls is rapidly decreased by borate ester cross-linking of the pectic polysaccharide rhamnogalacturonan II. *Plant Physiol.* 121 829–838. 10.1104/pp.121.3.829 10557231 PMC59445

[B45] Funck D. Baumgarten L. Stift M. von Wirén N. Schönemann L. (2020). Differential contribution of P5CS isoforms to stress tolerance in *Arabidopsis*. *Front. Plant Sci.* 11:565134. 10.3389/fpls.2020.565134 33101333 PMC7545825

[B46] Gao Y. Long R. Kang J. Wang Z. Zhang T. Sun H. (2019). Comparative proteomic analysis reveals that antioxidant system and soluble sugar metabolism contribute to salt tolerance in alfalfa (*Medicago sativa* L.) leaves. *J. Proteome Res.* 18 191–203. 10.1021/acs.jproteome.8b00521 30359026

[B47] Geng Y. Wu R. Wee C. W. Xie F. Wei X. Chan P. M. Y. (2013). A spatio-temporal understanding of growth regulation during the salt stress response in *Arabidopsis*. *Plant Cell* 25 2132–2154. 10.1105/tpc.113.112896 23898029 PMC3723617

[B48] Gong Q. Li P. Ma S. Indu Rupassara S. Bohnert H. J. (2005). Salinity stress adaptation competence in the extremophile *Thellungiella halophila* in comparison with its relative *Arabidopsis thaliana*. *Plant J.* 44 826–839. 10.1111/j.1365-313X.2005.02587.x 16297073

[B49] Gould K. S. Markham K. R. Smith R. H. Goris J. J. (2000). Functional role of anthocyanins in the leaves of *Quintinia serrata* A. Cunn. *J. Exp. Bot.* 51 1107–1115. 10.1093/jexbot/51.347.1107 10948238

[B50] Gupta B. Huang B. (2014). Mechanism of salinity tolerance in plants: physiological, biochemical, and molecular characterization. *Int. J. Genomics* 2014:701596. 10.1155/2014/701596 24804192 PMC3996477

[B51] Gupta D. K. Palma J. M. Corpas F. J. (2018). *Antioxidants and Antioxidant Enzymes in Higher Plants.* Cham: Springer.

[B52] Hildebrandt T. M. Nunes Nesi A. Araújo W. L. Braun H.-P. (2015). Amino acid catabolism in plants. *Mol. Plant* 8 1563–1579. 10.1016/j.molp.2015.09.005 26384576

[B53] Horie T. Hauser F. Schroeder J. I. (2009). HKT transporter-mediated salinity resistance mechanisms in *Arabidopsis* and monocot crop plants. *Trends Plant Sci.* 14 660–668. 10.1016/j.tplants.2009.08.009 19783197 PMC2787891

[B54] Jadamba C. Kang K. Paek N.-C. Lee S. I. Yoo S.-C. (2020). Overexpression of rice Expansin7 (Osexpa7) confers enhanced tolerance to salt stress in rice. *Int. J. Mol. Sci.* 21:454. 10.3390/ijms21020454 31936829 PMC7013816

[B55] Jahnke L. S. Hull M. R. Long S. P. (1991). Chilling stress and oxygen metabolizing enzymes in *Zea mays* and *Zea diploperennis*. *Plant Cell Environ.* 14 97–104. 10.1111/j.1365-3040.1991.tb01375.x

[B56] Jamet E. Albenne C. Boudart G. Irshad M. Canut H. Pont-Lezica R. (2008). Recent advances in plant cell wall proteomics. *Proteomics* 8 893–908. 10.1002/pmic.200700938 18210371

[B57] Jha D. Shirley N. Tester M. Roy S. J. (2010). Variation in salinity tolerance and shoot sodium accumulation in *Arabidopsis* ecotypes linked to differences in the natural expression levels of transporters involved in sodium transport. *Plant Cell Environ.* 33 793–804. 10.1111/j.1365-3040.2009.02105.x 20040066

[B58] Jia X. Zhu Y. Hu Y. Zhang R. Cheng L. Zhu Z. (2019). Integrated physiologic, proteomic, and metabolomic analyses of *Malus halliana* adaptation to saline–alkali stress. *Hortic. Res.* 6:91. 10.1038/s41438-019-0172-0 31645949 PMC6804568

[B59] Julkowska M. Hoefsloot H. C. Mol S. Feron R. de Boer G. J. Haring M. A. (2014). Capturing *Arabidopsis* root architecture dynamics with root-fit reveals diversity in responses to salinity. *Plant Physiol.* 166 1387–1402. 10.1104/pp.114.248963 25271266 PMC4226346

[B60] Julkowska M. Klei K. Fokkens L. Haring M. A. Schranz M. E. Testerink C. (2016). Natural variation in rosette size under salt stress conditions corresponds to developmental differences between *Arabidopsis* accessions and allelic variation in the LRR-KISS gene. *J. Exp. Bot.* 67 2127–2138. 10.1093/jxb/erw015 26873976 PMC4809279

[B61] Julkowska M. M. Koevoets I. T. Mol S. Hoefsloot H. Feron R. Tester M. A. (2017). Genetic components of root architecture remodeling in response to salt stress. *Plant Cell* 29 3198–3213. 10.1105/tpc.16.00680 29114015 PMC5757256

[B62] Katori T. Ikeda A. Iuchi S. Kobayashi M. Shinozaki K. Maehashi K. (2010). Dissecting the genetic control of natural variation in salt tolerance of *Arabidopsis thaliana* accessions. *J. Exp. Bot.* 61 1125–1138. 10.1093/jxb/erp376 20080827 PMC2826654

[B63] Kempa S. Krasensky J. Dal Santo S. Kopka J. Jonak C. (2008). A central role of abscisic acid in stress-regulated carbohydrate metabolism. *PLoS One* 3:e3935. 10.1371/journal.pone.0003935 19081841 PMC2593778

[B64] Kim S. Hwang G. Lee S. Zhu J.-Y. Paik I. Nguyen T. T. (2017). High ambient temperature represses anthocyanin biosynthesis through degradation of HY5. *Front. Plant Sci.* 8:1787. 10.3389/fpls.2017.01787 29104579 PMC5655971

[B65] Knaupp M. Mishra K. B. Nedbal L. Heyer A. G. (2011). Evidence for a role of raffinose in stabilizing photosystem II during freeze-thaw cycles. *Planta* 234 477–486. 10.1007/s00425-011-1413-0 21533754

[B66] Kohorn B. D. Kohorn S. L. (2012). The cell wall-associated kinases, WAKs, as pectin receptors. *Front. Plant Sci.* 3:88. 10.3389/fpls.2012.00088 22639672 PMC3355716

[B67] Kosová K. Vítámvás P. Urban M. O. Prášil I. T. (2013). Plant proteome responses to salinity stress – comparison of glycophytes and halophytes. *Funct. Plant Biol.* 40 775–786. 10.1071/FP12375 32481150

[B68] Kovinich N. Kayanja G. Chanoca A. Riedl K. Otegui M. S. Grotewold E. (2014). Not all anthocyanins are born equal: distinct patterns induced by stress in *Arabidopsis*. *Planta* 240 931–940. 10.1007/s00425-014-2079-1 24903357 PMC4200348

[B69] Krinsky N. I. (1979). Carotenoid protection against oxidation. *Pure Appl. Chem.* 51 649–660. 10.1351/pac197951030649

[B70] Kruskal W. H. Wallis W. A. (1952). Use of ranks in one-criterion variance analysis. *J. Am. Stat. Assoc.* 47 583–621. 10.2307/2280779

[B71] Kunz H.-H. Gierth M. Herdean A. Satoh-Cruz M. Kramer D. M. Spetea C. (2014). Plastidial transporters KEA1, -2, and -3 are essential for chloroplast osmoregulation, integrity, and pH regulation in *Arabidopsis*. *Proc. Natl. Acad. Sci. U.S.A.* 111 7480–7485. 10.1073/pnas.1323899111 24794527 PMC4034250

[B72] Kusano T. Berberich T. Tateda C. Takahashi Y. (2008). Polyamines: essential factors for growth and survival. *Planta* 228 367–381. 10.1007/s00425-008-0772-7 18594857

[B73] Kwon Y. R. Lee H. J. Kim K. H. Hong S.-W. Lee S. J. Lee H. (2008). Ectopic expression of *Expansin3* or *Expansinβ1* causes enhanced hormone and salt stress sensitivity in *Arabidopsis*. *Biotechnol. Lett.* 30 1281–1288. 10.1007/s10529-008-9678-5 18317696

[B74] Landi M. Tattini M. Gould K. S. (2015). Multiple functional roles of anthocyanins in plant-environment interactions. *Environ. Exp. Bot.* 119 4–17. 10.1016/j.envexpbot.2015.05.012

[B75] Le Gall H. Philippe F. Domon J.-M. Gillet F. Pelloux J. Rayon C. (2015). Cell wall metabolism in response to abiotic stress. *Plants* 4 112–166. 10.3390/plants4010112 27135320 PMC4844334

[B76] Leschevin M. Marcelo P. Ismael M. San-Clemente H. Jamet E. Rayon C. (2021). A Tandem Mass Tags (TMTs) labeling approach highlights differences between the shoot proteome of two *Arabidopsis thaliana* ecotypes, Col-0 and Ws. *Proteomics* e2000293. 10.1002/pmic.202000293 33891803

[B77] Leucci M. R. Lenucci M. S. Piro G. Dalessandro G. (2008). Water stress and cell wall polysaccharides in the apical root zone of wheat cultivars varying in drought tolerance. *J. Plant Physiol.* 165 1168–1180. 10.1016/j.jplph.2007.09.006 18155804

[B78] Li Y. Schellhorn H. E. (2007). Rapid kinetic microassay for catalase activity. *J. Biomol. Tech.* 18 185–187. 17916790 PMC2062561

[B79] Liu E. MacMillan C. P. Shafee T. Ma Y. Ratcliffe J. van de Meene A. (2020). Fasciclin-like arabinogalactan-protein 16 (FLA16) is required for stem development in *Arabidopsis*. *Front. Plant Sci.* 11:615392. 10.3389/fpls.2020.615392 33362841 PMC7758453

[B80] Liu J. Zhu J. K. (1997). Proline accumulation and salt-stress-induced gene expression in a salt-hypersensitive mutant of *Arabidopsis*. *Plant Physiol.* 114 591–596. 10.1104/pp.114.2.591 9193091 PMC158341

[B81] Lü P. Kang M. Jiang X. Dai F. Gao J. Zhang C. (2013). *RhEXPA4*, a rose expansin gene, modulates leaf growth and confers drought and salt tolerance to *Arabidopsis*. *Planta* 237 1547–1559. 10.1007/s00425-013-1867-3 23503758

[B82] Ma J. Wang D. She J. Li J. Zhu J.-K. She Y.-M. (2016). Endoplasmic reticulum-associated *N*-glycan degradation of cold-upregulated glycoproteins in response to chilling stress in *Arabidopsis*. *New Phytol.* 212 282–296. 10.1111/nph.14014 27558752 PMC5513495

[B83] Maathuis F. J. M. Ahmad I. Patishtan J. (2014). Regulation of Na^+^ fluxes in plants. *Front. Plant Sci.* 5:467. 10.3389/fpls.2014.00467 25278946 PMC4165222

[B84] Mäser P. Hosoo Y. Goshima S. Horie T. Eckelman B. Yamada K. (2002). Glycine residues in potassium channel-like selectivity filters determine potassium selectivity in four-loop-per-subunit HKT transporters from plants. *Proc. Natl. Acad. Sci. U.S.A.* 99 6428–6433. 10.1073/pnas.082123799 11959905 PMC122965

[B85] McGaughey S. A. Qiu J. Tyerman S. D. Byrt C. S. (2018). Regulating root aquaporin function in response to changes in salinity. *Annu. Plant Rev. Online* 1 381–416. 10.1002/9781119312994.apr0626

[B86] Miller G. Suzuki N. Ciftci-Yilmaz S. Mittler R. (2010). Reactive oxygen species homeostasis and signalling during drought and salinity stresses. *Plant Cell Environ.* 33 453–467. 10.1111/j.1365-3040.2009.02041.x 19712065

[B87] Minic Z. Rihouey C. Do C. T. Lerouge P. Jouanin L. (2004). Purification and characterization of enzymes exhibiting β-D-xylosidase activities in stem tissues of *Arabidopsis*. *Plant Physiol.* 135 867–878. 10.1104/pp.104.041269 15181203 PMC514122

[B88] Mitchell-Olds T. Schmitt J. (2006). Genetic mechanisms and evolutionary significance of natural variation in *Arabidopsis*. *Nature* 441 947–952. 10.1038/nature04878 16791187

[B89] Mittler R. (2002). Oxidative stress, antioxidants and stress tolerance. *Trends Plant Sci.* 7 405–410. 10.1016/s1360-1385(02)02312-9 12234732

[B90] Møller I. M. Jensen P. E. Hansson A. (2007). Oxidative modifications to cellular components in plants. *Annu. Rev. Plant Biol.* 58 459–481. 10.1146/annurev.arplant.58.032806.103946 17288534

[B91] Monroe J. D. Storm A. R. Badley E. M. Lehman M. D. Platt S. M. Saunders L. K. (2014). β-Amylase1 and β-amylase3 are plastidic starch hydrolases in *Arabidopsis* that seem to be adapted for different thermal, pH, and stress conditions. *Plant Physiol.* 166 1748–1763. 10.1104/pp.114.246421 25293962 PMC4256876

[B92] Mostofa M. G. Saegusa D. Fujita M. Tran L.-S. P. (2015). Hydrogen sulfide regulates salt tolerance in rice by maintaining Na^+^/K^+^ balance, mineral homeostasis and oxidative metabolism under excessive salt stress. *Front. Plant Sci.* 6:1055. 10.3389/fpls.2015.01055 26734015 PMC4685665

[B93] Munns R. Tester M. (2008). Mechanisms of salinity tolerance. *Annu. Rev. Plant Biol.* 59 651–681. 10.1146/annurev.arplant.59.032607.092911 18444910

[B94] Nishizawa A. Yabuta Y. Shigeoka S. (2008). Galactinol and raffinose constitute a novel function to protect plants from oxidative damage. *Plant Physiol.* 147 1251–1263. 10.1104/pp.108.122465 18502973 PMC2442551

[B95] Novaković L. Guo T. Bacic A. Sampathkumar A. Johnson K. L. (2018). Hitting the wall-sensing and signaling pathways involved in plant cell wall remodeling in response to abiotic stress. *Plants (Basel)* 7:89. 10.3390/plants7040089 30360552 PMC6313904

[B96] Olmos E. García De La Garma J. Gomez-Jimenez M. C. Fernandez-Garcia N. (2017). Arabinogalactan proteins are involved in salt-adaptation and vesicle trafficking in tobacco by-2 cell cultures. *Front. Plant Sci.* 8:1092. 10.3389/fpls.2017.01092 28676820 PMC5476920

[B97] Polle A. Chakrabarti K. Schürmann W. Renneberg H. (1990). Composition and properties of hydrogen peroxide decomposing systems in extracellular and total extracts from needles of Norway spruce (*Picea abies* L., Karst.) 1. *Plant Physiol.* 94 312–319. 10.1104/pp.94.1.312 16667703 PMC1077226

[B98] Pontarin N. Molinié R. Mathiron D. Tchoumtchoua J. Bassard S. Gagneul D. (2020). Age-dependent metabolic profiles unravel the metabolic relationships within and between flax leaves (*Linum usitatissimum*). *Metabolites* 10:218. 10.3390/metabo10060218 32466546 PMC7345097

[B99] Pritchard S. G. Ju Z. Van Santen E. Qiu J. Weaver D. B. Prior S. A. (2000). The influence of elevated CO_2_ on the activities of antioxidative enzymes in two soybean genotypes. *Austrian J. Plant Physiol.* 27 1061–1068. 10.1071/PP99206

[B100] Quéro A. Béthencourt L. Pilard S. Fournet A. Guillot X. X. Sangwan R. (2013). Trehalose determination in linseed subjected to osmotic stress. HPAEC-PAD analysis: an inappropriate method. *Physiol. Plant.* 147 261–269. 10.1111/j.1399-3054.2012.01677.x 22901048

[B101] Rabino I. Mancinelli A. L. (1986). Light, temperature, and anthocyanin production. *Plant Physiol.* 81 922–924. 10.1104/pp.81.3.922 16664926 PMC1075451

[B102] Roy S. J. Negrão S. Tester M. (2014). Salt resistant crop plants. *Curr. Opin. Biotechnol.* 26 115–124. 10.1016/j.copbio.2013.12.004 24679267

[B103] Roychoudhury A. Basu S. Sengupta D. N. (2011). Amelioration of salinity stress by exogenously applied spermidine or spermine in three varieties of indica rice differing in their level of salt tolerance. *J. Plant Physiol.* 168 317–328. 10.1016/j.jplph.2010.07.009 20728960

[B104] Ruiz-Sola M. Á Arbona V. Gómez-Cadenas A. Rodríguez-Concepción M. Rodríguez-Villalón A. (2014). A root specific induction of carotenoid biosynthesis contributes to ABA production upon salt stress in *Arabidopsis*. *PLoS One* 9:e90765. 10.1371/journal.pone.0090765 24595399 PMC3942475

[B105] Rus A. Baxter I. Muthukumar B. Gustin J. Lahner B. Yakubova E. (2006). Natural variants of AtHKT1 enhance Na^+^ accumulation in two wild populations of *Arabidopsis*. *PLoS Genet.* 2:e210. 10.1371/journal.pgen.0020210 17140289 PMC1665649

[B106] Saha J. Brauer E. K. Sengupta A. Popescu S. C. Gupta K. Gupta B. (2015). Polyamines as redox homeostasis regulators during salt stress in plants. *Front. Environ. Sci.* 3:21. 10.3389/fenvs.2015.00021

[B107] San Clemente H. Pont-Lezica R. Jamet E. (2009). Bioinformatics as a tool for assessing the quality of sub-cellular proteomic strategies and inferring functions of proteins: plant cell wall proteomics as a test case. *Bioinform. Biol. Insights* 3 15–28. 10.4137/bbi.s2065 20140071 PMC2808182

[B108] Santelia D. Lunn J. E. (2017). Transitory starch metabolism in guard cells: unique features for a unique function. *Plant Physiol.* 174 539–549. 10.1104/pp.17.00211 28292855 PMC5462065

[B109] Schneider T. Keller F. (2009). Raffinose in chloroplasts is synthesized in the cytosol and transported across the chloroplast envelope. *Plant Cell Physiol.* 50 2174–2182. 10.1093/pcp/pcp151 19880397

[B110] Seifert G. J. (2004). Nucleotide sugar interconversions and cell wall biosynthesis: how to bring the inside to the outside. *Curr. Opin. Plant Biol.* 7 277–284. 10.1016/j.pbi.2004.03.004 15134748

[B111] Seifert G. J. Xue H. Acet T. (2014). The *Arabidopsis thaliana FASCICLIN LIKE ARABINOGALACTAN PROTEIN 4* gene acts synergistically with abscisic acid signalling to control root growth. *Ann. Bot.* 114 1125–1133. 10.1093/aob/mcu010 24603604 PMC4195540

[B112] Shabala S. Demidchik V. Shabala L. Cuin T. A. Smith S. J. Miller A. J. (2006). Extracellular Ca^2+^ ameliorates NaCl^–^induced K^+^ loss from *Arabidopsis* root and leaf cells by controlling plasma membrane K^+^-permeable channels. *Plant Physiol.* 141 1653–1665. 10.1104/pp.106.082388 16798942 PMC1533937

[B113] Shafi A. Gill T. Zahoor I. Ahuja P. S. Sreenivasulu Y. Kumar S. (2019). Ectopic expression of *SOD* and *APX* genes in *Arabidopsis* alters metabolic pools and genes related to secondary cell wall cellulose biosynthesis and improve salt tolerance. *Mol. Biol. Rep.* 46 1985–2002. 10.1007/s11033-019-04648-3 30706357

[B114] Sharma A. Shahzad B. Kumar V. Kohli S. K. Sidhu G. P. S. Bali A. S. (2019). Phytohormones regulate accumulation of osmolytes under abiotic stress. *Biomolecules* 9:285. 10.3390/biom9070285 31319576 PMC6680914

[B115] Shi H. Lee B. Wu S.-J. Zhu J.-K. (2003). Overexpression of a plasma membrane Na^+^/H^+^ antiporter gene improves salt tolerance in *Arabidopsis thaliana*. *Nat. Biotechnol.* 21 81–85. 10.1038/nbt766 12469134

[B116] Singh V. Singh A. P. Bhadoria J. Giri J. Singh J. Vineeth T. V. (2018). Differential expression of salt-responsive genes to salinity stress in salt-tolerant and salt-sensitive rice (*Oryza sativa* L.) at seedling stage. *Protoplasma* 255 1667–1681. 10.1007/s00709-018-1257-6 29740721

[B117] Slama I. Abdelly C. Bouchereau A. Flowers T. Savouré A. (2015). Diversity, distribution and roles of osmoprotective compounds accumulated in halophytes under abiotic stress. *Ann. Bot.* 115 433–447. 10.1093/aob/mcu239 25564467 PMC4332610

[B118] Stephan A. B. Kunz H.-H. Yang E. Schroeder J. I. (2016). Rapid hyperosmotic-induced Ca2+ responses in *Arabidopsis thaliana* exhibit sensory potentiation and involvement of plastidial KEA transporters. *Proc. Natl. Acad. Sci. U.S.A.* 113 E5242–E5249. 10.1073/pnas.1519555113 27528686 PMC5024618

[B119] Stepien P. Johnson G. N. (2009). Contrasting responses of photosynthesis to salt stress in the glycophyte *Arabidopsis* and the halophyte *Thellungiella*: role of the plastid terminal oxidase as an alternative electron sink. *Plant Physiol.* 149 1154–1165. 10.1104/pp.108.132407 19052149 PMC2633845

[B120] Sun J. Dai S. Wang R. Chen S. Li N. Zhou X. (2009). Calcium mediates root K^+^/Na^+^ homeostasis in poplar species differing in salt tolerance. *Tree Physiol.* 29 1175–1186. 10.1093/treephys/tpp048 19638360

[B121] Sun Z. Qi X. Wang Z. Li P. Wu C. Zhang H. (2013). Overexpression of *TsGOLS2*, a galactinol synthase, in *Arabidopsis thaliana* enhances tolerance to high salinity and osmotic stresses. *Plant Physiol. Biochem.* 69 82–89. 10.1016/j.plaphy.2013.04.009 23728391

[B122] Thalmann M. Pazmino D. Seung D. Horrer D. Nigro A. Meier T. (2016). Regulation of leaf starch degradation by abscisic acid is important for osmotic stress tolerance in plants. *Plant Cell* 28 1860–1878. 10.1105/tpc.16.00143 27436713 PMC5006701

[B123] Thalmann M. Santelia D. (2017). Starch as a determinant of plant fitness under abiotic stress. *New Phytol.* 214 943–951. 10.1111/nph.14491 28277621

[B124] Triantaphylidès C. Krischke M. Hoeberichts F. A. Ksas B. Gresser G. Havaux M. (2008). Singlet oxygen is the major reactive oxygen species involved in photooxidative damage to plants. *Plant Physiol.* 148 960–968. 10.1104/pp.108.125690 18676660 PMC2556806

[B125] Tsujii M. Kera K. Hamamoto S. Kuromori T. Shikanai T. Uozumi N. (2019). Evidence for potassium transport activity of *Arabidopsis* KEA1-KEA6. *Sci. Rep.* 9:10040. 10.1038/s41598-019-46463-7 31296940 PMC6624313

[B126] Uehlein N. Sperling H. Heckwolf M. Kaldenhoff R. (2012). The *Arabidopsis* aquaporin PIP1;2 rules cellular CO_2_ uptake. *Plant Cell Environ.* 35 1077–1083. 10.1111/j.1365-3040.2011.02473.x 22150826

[B127] van Oosten M. J. Sharkhuu A. Batelli G. Bressan R. A. Maggio A. (2013). The *Arabidopsis thaliana* mutant air1 implicates SOS3 in the regulation of anthocyanins under salt stress. *Plant Mol. Biol.* 83 405–415. 10.1007/s11103-013-0099-z 23925404

[B128] Wang X. Chang L. Wang B. Wang D. Li P. Wang L. (2013). Comparative proteomics of *Thellungiella halophila* leaves from plants subjected to salinity reveals the importance of chloroplastic starch and soluble sugars in halophyte salt tolerance. *Mol. Cell. Proteomics* 12 2174–2195. 10.1074/mcp.M112.022475 23660471 PMC3734578

[B129] Ward J. L. Baker J. M. Llewellyn A. M. Hawkins N. D. Beale M. H. (2011). Metabolomic analysis of *Arabidopsis* reveals hemiterpenoid glycosides as products of a nitrate ion-regulated, carbon flux overflow. *Proc. Natl. Acad. Sci. U.S.A.* 108 10762–10767. 10.1073/pnas.1018875108 21670294 PMC3127892

[B130] Wu D. Cai S. Chen M. Ye L. Chen Z. Zhang H. (2013). Tissue metabolic responses to salt stress in wild and cultivated barley. *PLoS One* 8:e55431. 10.1371/journal.pone.0055431 23383190 PMC3561194

[B131] Yan J. He H. Fang L. Zhang A. (2018). Pectin methylesterase31 positively regulates salt stress tolerance in *Arabidopsis*. *Biochem. Biophys. Res. Commun.* 496 497–501. 10.1016/j.bbrc.2018.01.025 29307824

[B132] Yan J. Huang Y. He H. Han T. Di P. Sechet J. (2019). Xyloglucan endotransglucosylase-hydrolase30 negatively affects salt tolerance in *Arabidopsis*. *J. Exp. Bot.* 70 5495–5506. 10.1093/jxb/erz311 31257449 PMC6793456

[B133] Zapata P. J. Serrano M. García-Legaz M. F. Pretel M. T. Botella M. A. (2017). Short term effect of salt shock on ethylene and polyamines depends on plant salt sensitivity. *Front. Plant Sci.* 8:855. 10.3389/fpls.2017.00855 28588603 PMC5440749

[B134] Zhang Q. Su L.-J. Chen J.-W. Zeng X.-Q. Sun B.-Y. Peng C.-L. (2012). The antioxidative role of anthocyanins in *Arabidopsis* under high-irradiance. *Biol. Plant.* 56 97–104. 10.1007/s10535-012-0022-5

[B135] Zhao C. Zayed O. Yu Z. Jiang W. Zhu P. Hsu C.-C. (2018). Leucine-rich repeat extensin proteins regulate plant salt tolerance in *Arabidopsis*. *Proc. Natl. Acad. Sci. U.S.A.* 115 13123–13128. 10.1073/pnas.1816991115 30514814 PMC6305001

[B136] Zhao C. Zayed O. Zeng F. Liu C. Zhang L. Zhu P. (2019). Arabinose biosynthesis is critical for salt stress tolerance in *Arabidopsis*. *New Phytol.* 224 274–290. 10.1111/nph.15867 31009077

