# Supplementary material for: Physiological and Biochemical Traits of Two Major Arabidopsis Accessions, Col-0 and Ws, Under Salinity
Source: Front Plant Sci. 2021 Jun 21;12:639154. doi: 10.3389/fpls.2021.639154 (PMC8256802; doi:10.3389/fpls.2021.639154)
Supplement: Supplementary Figure 1 — Shoot biomass and shoot tolerance index. [file Data_Sheet_2.pdf]

Physiological and biochemical traits of two major Arabidopsis accessions, Col-0 and Ws, under salinity

Leschevin *et al.*

Fig. S1. Effect on salt stress on shoot biomass (A) and (B) shoot tolerance index (TI)

Fig. S2. Photosynthetic pigment content ($mg.g^{-1}$ DM) in Ws and Col-0 control and salt-treated plants

Fig. S3. Enzymatic activities of oxido-reductases in Ws and Col-0 control and salt-treated plants

Fig. S1. Shoot biomass and shoot tolerance index. (A) Shoots fresh mass and (B) shoot tolerance index in plants grown under control conditions and 150 mM of NaCl at T48, T72 and T96. Data are means ± standard deviation (SD) of three biological replicates. Means followed by the same letter in the same graph are not significantly different according to Dunn test.

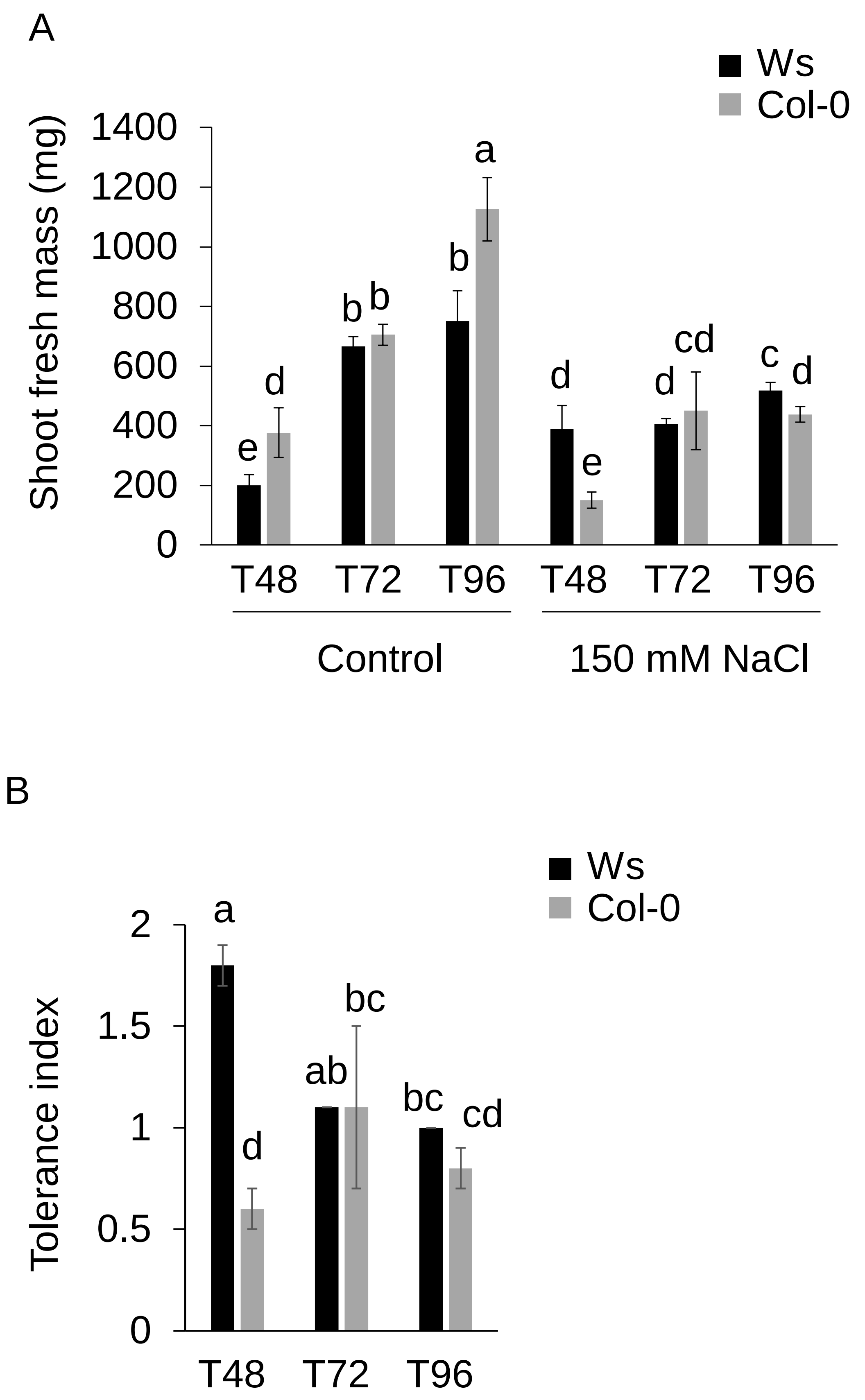

Fig. S2. Photosynthetic pigment content (mg.g^{-1} DM) in Ws and Col-0 control and salt-treated plants. (A) Chlorophyll and (B) carotenoid contents in plants grown under control conditions and 150 mM of NaCl at T48, T72 and T96. Chl meaning chlorophyll, xanthophyll being the sum of lutein, neoxanthin, violaxanthin and antheraxanthin. Data are means ± standard deviation (SD) of three biological replicates. Means followed by the same letter for the same pigment in the same graph are not significantly different according to Dunn test.

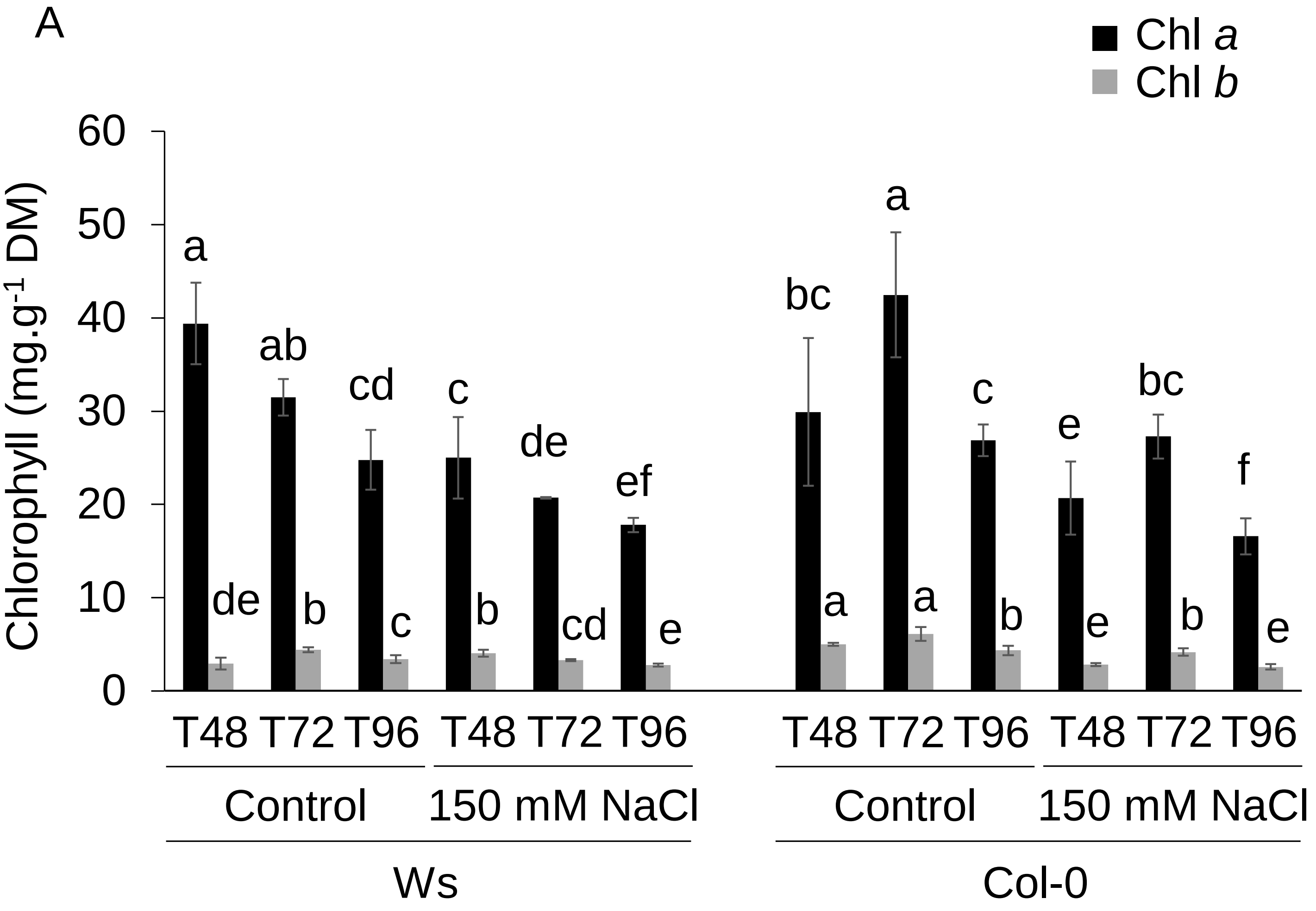

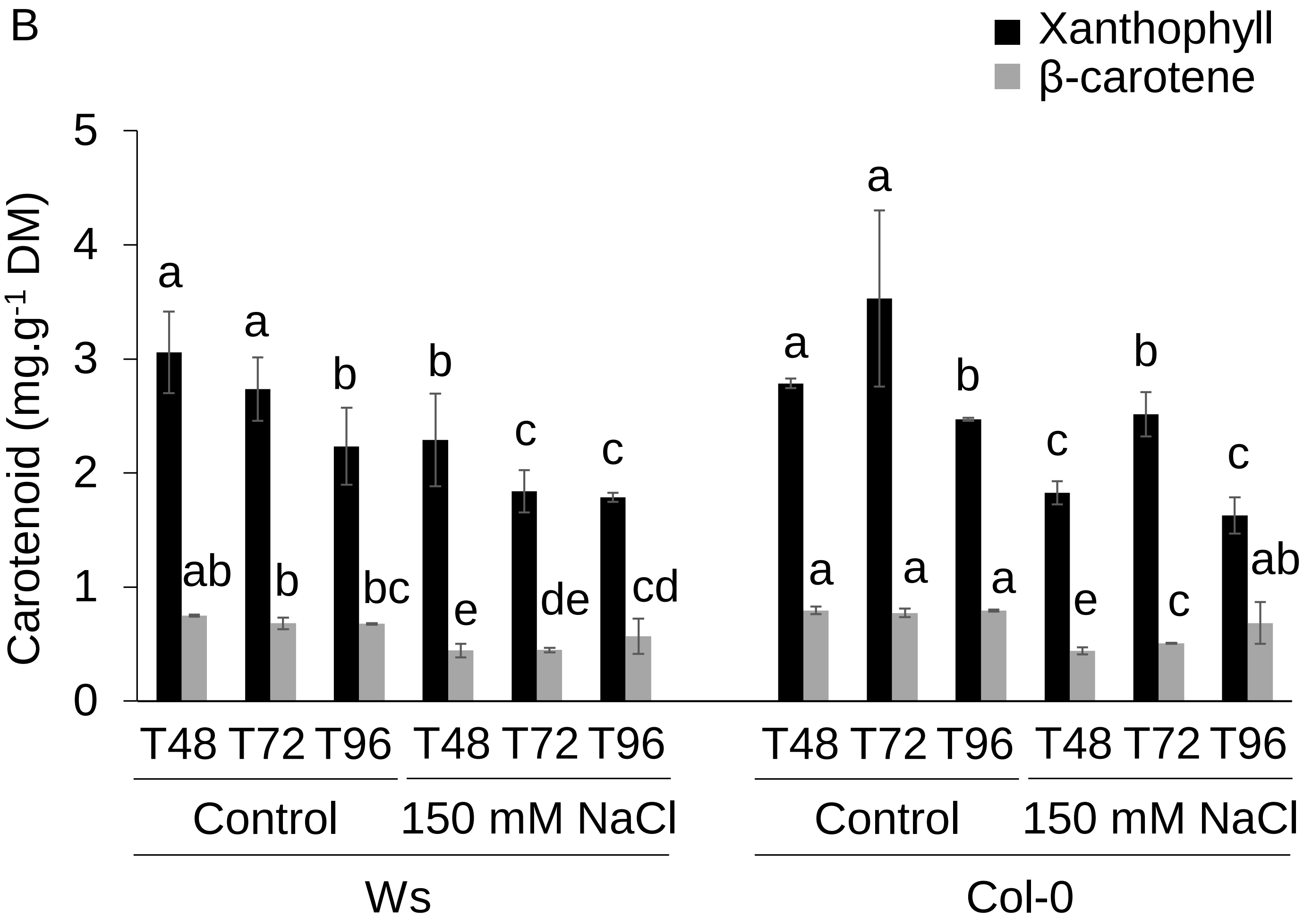
B
Xanthophyll
β-carotene
Carotenoid (mg.g-1 DM)
5
4
3
2
1
0
a
a
b
b
c
c
ab
b
bc
e
de
cd
a
a
b
c
b
c
a
a
a
e
c
ab
T48 T72 T96 T48 T72 T96
Control
150 mM NaCl
Ws
T48 T72 T96 T48 T72 T96
Control
150 mM NaCl
Col-0

Fig. S3. Enzymatic activities of oxido-reductases in Ws and Col-0 control and salt-treated plants. (A) CAT, (B) APOX and (C) GR enzyme activities measured under control conditions and 150 mM of NaCl at T48, T72 and T96. CAT stands for catalase, APOX for ascorbate peroxidase and GR for glutathione reductase. Data are means ± standard deviation (SD) of three biological replicates. Means followed by the same letter in the same graph are not significantly different according to Dunn test.

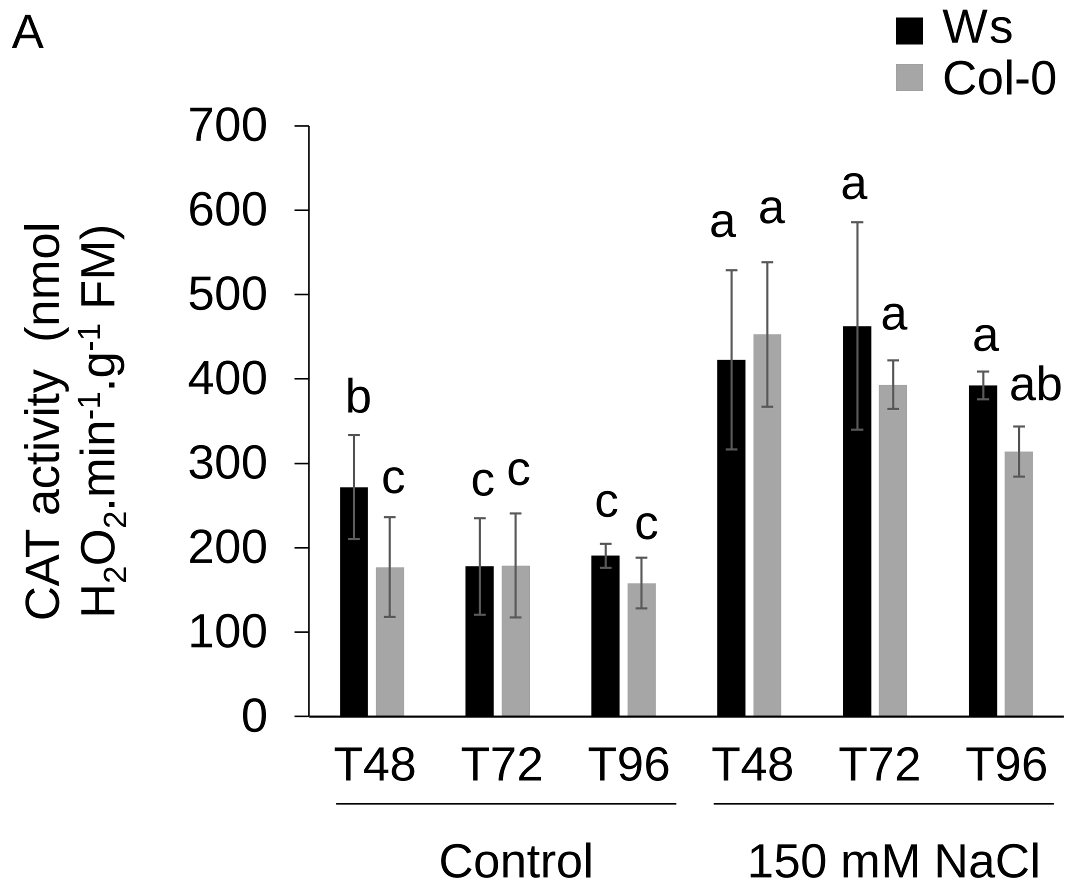

B

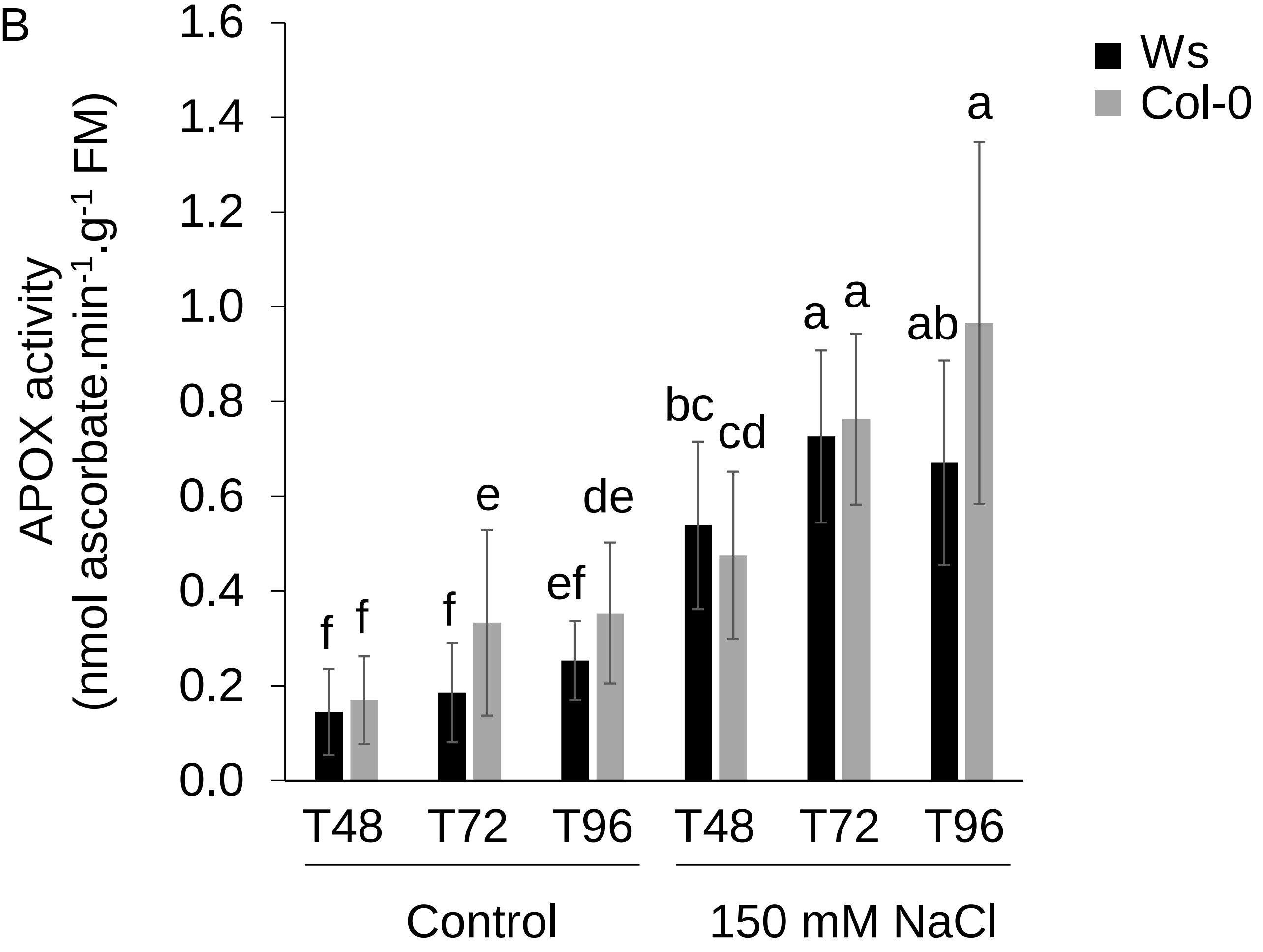

APOX activity
(nmol ascorbate.min-1.g-1 FM)
1.6
1.4
1.2
1.0
0.8
0.6
0.4
0.2
0.0
Ws
Col-0
f
f
f
e
ef
de
bc
cd
a
a
ab
a
T48
T72
T96
T48
T72
T96
Control
150 mM NaCl

C

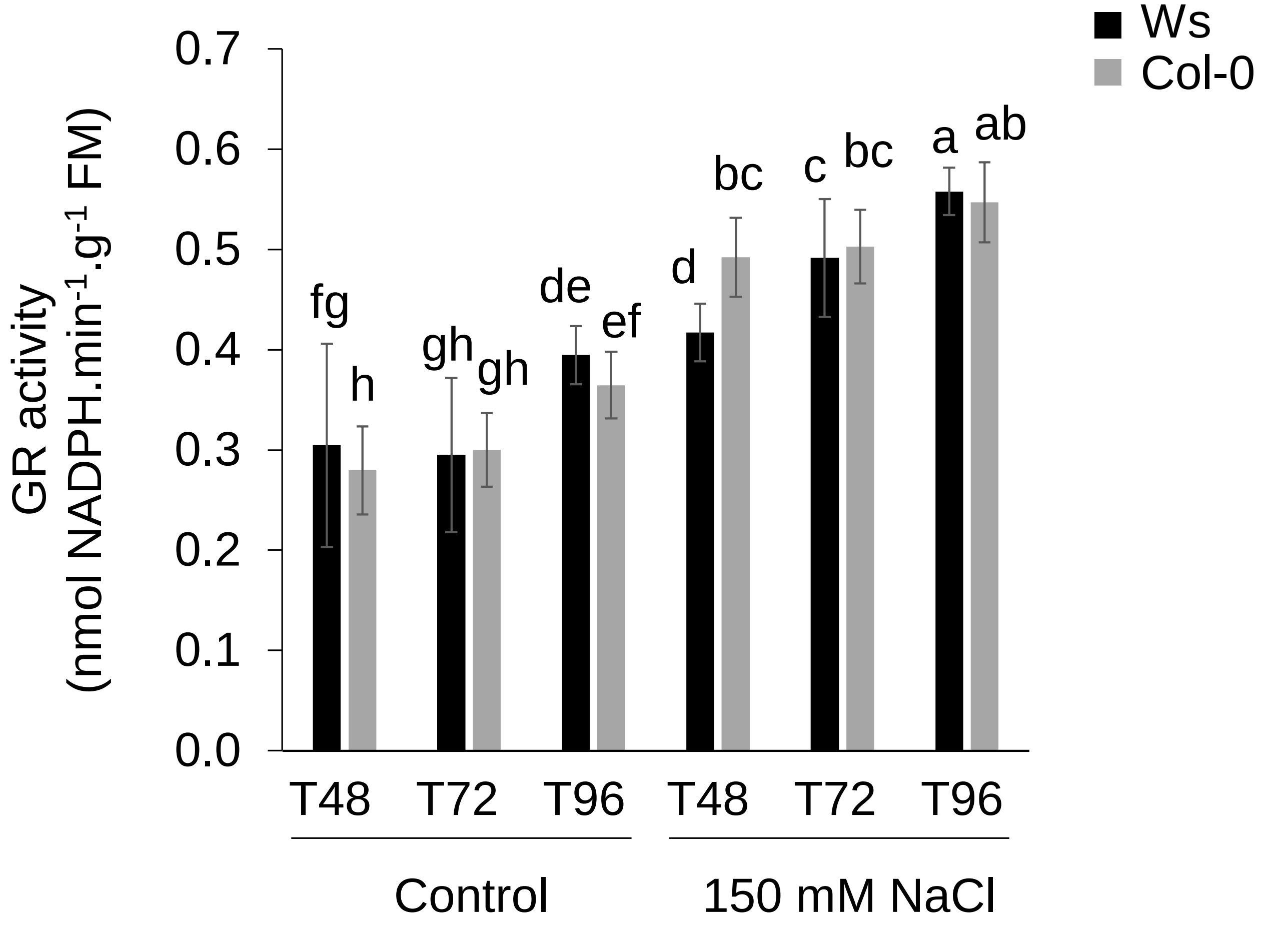

GR activity
(nmol NADPH.min-1.g-1 FM)
0.7
0.6
0.5
0.4
0.3
0.2
0.1
0.0
Ws
Col-0
fg
h
gh
gh
de
ef
d
bc
c
bc
a
ab
T48
T72
T96
T48
T72
T96
Control
150 mM NaCl
