# Supplementary material for: Physiological and Biochemical Traits of Two Major Arabidopsis Accessions, Col-0 and Ws, Under Salinity
Source: Front Plant Sci. 2021 Jun 21;12:639154. doi: 10.3389/fpls.2021.639154 (PMC8256802; doi:10.3389/fpls.2021.639154)
Supplement: Supplementary file 8 [file Data_Sheet_1.docx]

**Supplementary Information for**

**Physiological and biochemical traits of two major Arabidopsis accessions, Col-0 and Ws, under salinity**

**Maïté Leschevin^1^, Marwa Ismael^1^, Anthony Quero^1^, Hélène San Clemente^2^, Romain Roulard^1^, Solène Bassard^1^, Paulo Marcelo^3^, Karine Pageau^1^, Elisabeth Jamet^2^, and Catherine Rayon^1*^**

**Materials and Methods**

**Antioxidant Enzymatic Activity Assays**

Proteins were extracted from 20 mg of frozen shoot powder in 500 µL of cold potassium phosphate buffer (0.1 M, pH 7.5) containing polyvinylpyrrolidone 1%. The homogenate was centrifuged at 4°C for 10 min at 14,000 *g*. The supernatant was used for the assay of antioxidative enzymatic activities (SOD, CAT), and enzymes involved in the ascorbate-glutathione cycle (APX, MDHAR, DHAR and GR).

All the assays were performed in 96 well-microplates and absorbances were read in a microplate reader (Powerwave, Biotek, Colmar, France).

- A continuous assay was used to measure the **SOD** enzymatic activity using an adapted method from Ewing and Janero (1995). The protein extract (25 µL) was added to a mix containing potassium phosphate (50 mM pH 7.4), 0.4 mM EDTA, 0.2 mM NBT, 0.3 mM NADH and the activity was measured for 10 min at 560 nm. The SOD enzymatic activity was assessed as a percentage of inhibition of O_2_^-^-dependent NBT reduction and expressed in SOD unit.g^-1^ FM with one SOD unit equal to 50% inhibition of O_2_^-^-dependent NBT reduction.

- A continuous assay was performed to measure the **CAT** anti-oxidant capacity according to Li and Schellhorn (2007), an assay based on the rate of decomposition of H_2_O_2_, which is proportional to the reduction of the absorbance at 240 nm. The protein extract (10 µL) was incubated in 250 µL of the reaction buffer (potassium phosphate buffer 50 mM, pH 7, 25 mM H_2_O_2_) for 6 min at RT. The CAT capacity was expressed in nmol of consumed H_2_O_2_.min^-1^.g^-1^ FM.

- The **APX** enzymatic activity was assayed according to the method adapted from Jahnke *et al.* (1991). It was measured by the rate of ascorbate oxidation at 290 nm. The reaction mixture (90 µL) containing potassium phosphate buffer 0.1 M pH 7, 0.5 mM ascorbate, 0.2 mM EDTA and 5 mM H_2_O_2_ was added to the protein extract (10 µL) and the activity was measured every 30 s for 5 min. The APX enzymatic activity was expressed in nmol of consumed ascorbate.min^-1^.g^-1^ FM.

- Methods adapted from (Pritchard *et al.*, 2000) were used to determine the **MDHAR** and **DHAR** capacities. The MDHAR enzymatic activity was assayed by coupling the protein extract to NADH, ascorbate and an excess of ascorbate oxidase to generate monodehydroascorbate and NADH oxidation. The protein extract (15 µL) was incubated with the reaction buffer (85 µL) containing potassium phosphate 0.1 M pH 7.5, 0.25 mM NADH, 1.5 mM ascorbate and 0.02 U of ascorbate oxidase for 5 min at RT. The absorbance was read every 30 s. MDHAR enzymatic activity corresponded to the rate of decrease in absorbance at 340 nm due to NADH oxidation expressed in nmol of consumed NADH.min^-1.^g^-1^ FM. DHAR enzymatic activity was determined by the rate of dehydroascorbate reduction. The reaction mixture (85 µL) containing potassium phosphate buffer 0.1 M pH 6.5, 0.5 mM EDTA, 2 mM reduced glutathione and 150 µM dehydroascorbate was added to the protein extract (15 µL) and incubated at RT. The sample was read every min during 30 min at 290 nm. DHAR activity was expressed in nmol of produced ascorbate.min^-1^.g^-1^ FM.

- The **GR** enzymatic activity was assayed following a method adapted from (Polle et al., 1990). The reaction mixture (85 µL) containing 50 mM Tricine pH 7.8, 0.5 mM EDTA, 0.25 mM oxidized glutathione and 0.2 mM NADPH was added to the protein extract (15 µL) and incubated for 30 min at room temperature. The decrease in absorbance at 340 nm was measured every min. GR enzymatic activity was determined by the rate of glutathione-dependent oxidation of NADPH. Control rates obtained in the absence of oxidized glutathione were subtracted to eliminate the non-enzymatic conversion. The GR enzymatic activity was expressed in nmol of consumed NADPH.min^-1^.g^-1^ FM.
